# Mitochondrial DNA control-region and coding-region data highlight geographically structured diversity and post-domestication population dynamics in worldwide donkeys

**DOI:** 10.1371/journal.pone.0307511

**Published:** 2024-08-28

**Authors:** Nicola Rambaldi Migliore, Daniele Bigi, Marco Milanesi, Paolo Zambonelli, Riccardo Negrini, Simone Morabito, Andrea Verini-Supplizi, Luigi Liotta, Fatima Chegdani, Saif Agha, Bashir Salim, Albano Beja-Pereira, Antonio Torroni, Paolo Ajmone‐Marsan, Alessandro Achilli, Licia Colli

**Affiliations:** 1 Dipartimento di Biologia e Biotecnologie “Lazzaro Spallanzani”, University of Pavia, Via A. Ferrata, Pavia, Italy; 2 Dipartimento di Scienze e Tecnologie Agro-Alimentari (DISTAL), Alma Mater Studiorum Università di Bologna, Viale Giuseppe Fanin, Bologna, Italy; 3 Dipartimento di Scienze Animali, della Nutrizione e degli Alimenti, Università Cattolica del S. Cuore, via Emilia Parmense, Piacenza, Italy; 4 Dipartimento di Medicina Veterinaria, University of Perugia, Perugia, Italy; 5 Department of Veterinary Sciences, University of Messina, V.le Palatucci, Messina, Italy; 6 Animal Production Department, Faculty of Agriculture, Ain Shams University, Cairo, Egypt; 7 Department of Parasitology, Faculty of Veterinary Medicine, University of Khartoum, Khartoum-North, Sudan; 8 Camel Research Center, King Faisal University, Al-Ahsa, Saudi Arabia; 9 CIBIO, Centro de Investigação em Biodiversidade e Recursos Genéticos, InBIO Laboratório Associado, Campus de Vairão, Universidade do Porto, Vairão, Portugal; 10 BIOPOLIS Program in Genomics, Biodiversity and Land Planning, CIBIO, Campus de Vairão, Vairão, Portugal; 11 DGAOT, Faculty of Sciences, Universidade do Porto, Rua Campo Alegre, Porto, Portugal; 12 CREI Romeo and Enrica Invernizzi Research Center on Sustainable Dairy Production, Università Cattolica del S. Cuore, Piacenza (PC), Italy; 13 BioDNA Centro di Ricerca Sulla Biodiversità e sul DNA Antico, Università Cattolica del S. Cuore, Piacenza (PC), Italy; University of Bari, ITALY

## Abstract

Donkeys (*Equus asinus*) have been used extensively in agriculture and transportations since their domestication, ca. 5000–7000 years ago, but the increased mechanization of the last century has largely spoiled their role as burden animals, particularly in developed countries. Consequently, donkey breeds and population sizes have been declining for decades, and the diversity contributed by autochthonous gene pools has been eroded. Here, we examined coding-region data extracted from 164 complete mitogenomes and 1392 donkey mitochondrial DNA (mtDNA) control-region sequences to (i) assess worldwide diversity, (ii) evaluate geographical patterns of variation, and (iii) provide a new nomenclature of mtDNA haplogroups. The topology of the Maximum Parsimony tree confirmed the two previously identified major clades, i.e. Clades 1 and 2, but also highlighted the occurrence of a deep-diverging lineage within Clade 2 that left a marginal trace in modern donkeys. Thanks to the identification of stable and highly diagnostic coding-region mutational motifs, the two lineages were renamed as haplogroup A and haplogroup B, respectively, to harmonize clade nomenclature with the standard currently adopted for other livestock species. Control-region diversity and population expansion metrics varied considerably between geographical areas but confirmed North-eastern Africa as the likely domestication center. The patterns of geographical distribution of variation analyzed through phylogenetic networks and AMOVA confirmed the co-occurrence of both haplogroups in all sampled populations, while differences at the regional level point to the joint effects of demography, past human migrations and trade following the spread of donkeys out of the domestication center. Despite the strong decline that donkey populations have undergone for decades in many areas of the world, the sizeable mtDNA variability we scored, and the possible identification of a new early radiating lineage further stress the need for an extensive and large-scale characterization of donkey nuclear genome diversity to identify hotspots of variation and aid the conservation of local breeds worldwide.

## Introduction

       *Orientis partibus/ adventavit asinus/ pulcher et fortissimus/ sarcinis aptissimus*.

         “From the East/ the donkey came/ strong and beautiful/ fit for burden”.

        “*Orientis partibus*”, Medieval song, Anonymous, 12th century ca.

Animal domestication was essential for the development of human civilizations and culture. Sheep, goats, cattle, and pigs were domesticated between 10 and 13 thousand years ago (kya–kilo years ago) and initially exploited as a readily available source of meat, and later for dairy production and traction. Animals used to carry burdens and people, such as horses, donkeys, and camels, were domesticated later, about 5–7 kya [1–3]. Evidence based on genetic studies and archaeological remains strongly supports an African origin of domestic donkeys, *Equus asinus* [1, 4–7]. In the most consensual view, based on the “pastoralist hypothesis”, donkeys were domesticated by herders 7.5–6.5 kya in the northeast African grasslands, within an area spanning from Egypt and the Nile Valley to the Western Red Sea and Eritrea, when unpredictable rainfalls and severe droughts followed a climatic deterioration and led to the increasing aridity of the Sahara, forcing human populations to adopt an increasingly mobile way of life [8–10].

A pioneeristic study based on mitochondrial DNA (mtDNA) control-region variation, carried out by Beja-Pereira et al. [4] on 427 donkeys from 52 countries across Africa and Eurasia, pinpointed the African wild ass (*Equus africanus*) as the ancestor species of the domestic donkey and revealed two distinct maternal lineages, termed Clade 1 and Clade 2, interpreted as evidence of two independent domestication events. Beja-Pereira et al. [4] and Kimura et al. [11] proposed that Clade 1 derived from the Nubian wild ass (*E*. *a*. *africanus*). In contrast, the origin of Clade 2 is less clear as Kimura et al. [11] excluded that this lineage could have arisen from the endangered Somali wild ass (*E*. *a*. *somaliensis*) and traced its origin back to a yet unrecognized extinct African wild ass population. The two clades have been described in almost all domestic donkey populations characterized so far, although with varying frequencies [4, 12]. Recently, the analysis of whole genome sequences of both modern and ancient donkeys from around the world [7] supported domestication ca. 7 kya from a unique African source population (in which both Clades 1 and 2 were already represented), followed by a spread to Eurasia starting around 4.5 kya, and by subsequent intra-continental diversification as well as gene flows events. The occurrence of divergent genetic material was also revealed, pointing at an ongoing introgression from African wild asses into modern donkeys from Africa/Southern Arabian Peninsula and contributions from kiangs (*Equus kiang*) and another divergent lineage to domestic donkeys from China and south-western Asia, respectively.

After the initial global survey by Beja-Pereira et al. [4] several studies have focused on the description of mtDNA sequence diversity of local donkey populations in specific geographic areas such as Croatia [13], Spain [14], Mexico [15], China [16, 17], Ethiopia [5], the Balkans [18], Turkey [19, 20], Serbia [21], Southern America [22] and Pakistan [23]. Ma et al. [12] performed a shallow analysis of worldwide donkey haplogroup distribution, but no detailed appraisal of the global mtDNA variation has been carried out for this species, so far.

Furthermore, the nomenclature used to identify donkey mtDNA haplogroups (i.e. Clades 1 and 2) is at odds with the alphanumerical coding system adopted for all other livestock species, which allows a more detailed description of lineages and sub-lineages [2, 24–26].

The growing body of knowledge on the molecular variation of livestock species and the analysis of its changes through time have shown that, due to the abrupt changes occurred in the agricultural practices during the last century, a large share of the original diversity has already disappeared, particularly in developed countries [27]. The donkey has been no exception to this trend. In fact, due to factors as the mechanization of agriculture and transports, a remarkable drop in donkey population sizes has been observed during the first half of the last century in several countries around the world [28]. Interestingly, the global number of donkeys has been rising by 1% annually since the 1960’s, with the largest increases and reductions being observed in sub-Saharan Africa and Eastern Europe, respectively. This trend indicates that the changes in donkey census sizes are influenced by a variety of socio-economic factors which vary by nation and region [29]. A global survey of the mtDNA variability is useful to map the worldwide distribution of diversity, and to identify hotspots of variations and evolutionary relationships between populations of different geographical areas. Therefore, the present study aims are to (i) carry out a global-scale analysis of domestic donkey mtDNA diversity based on a worldwide dataset encompassing 1392 control-region sequences (374 new and 1018 from previous studies), (ii) evaluate the patterns of variation in different geographical and historical contexts, and (iii) to propose a new nomenclature for donkey mtDNA haplogroups harmonized with other livestock.

## Methods

### Collection of biological samples and DNA extraction

A total of 341 Italian donkeys, from eight breeds [Asinara (ASI, n = 64), Sardo (SAR, n = 43), Amiata (AMI, n = 46), Grigio Siciliano (GRS, n = 30), Martina Franca (MAF, n = 31) Pantesco (PAN, n = 30), Ragusano (RAG, n = 40) and Romagnolo (ROM, n = 28)] and from donkeys not assigned to any breed, Unknown Breed (UNK, n = 29), were analyzed to obtain mtDNA control-region sequences. The samples have been collected as part of a project aiming at the characterization of the diversity of Italian native donkey breeds. The selection of the individuals was carried out so to minimize the relatedness and to include both males and females approximately in a 1:3 ratio. The geographical distribution of the sampled breeds over the Italian territory and their variation at microsatellite loci is described in Colli et al. [30]. Additionally, mtDNA sequence data of 33 African *E*. *asinus* from four different locations [Egypt (EGY, n = 9), Sudan (SUD, n = 6), Morocco (MOR, n = 16) and Tanzania (TAN, n = 2)] was also assessed.

For the ASI, SAR, GRS, PAN, RAG and UNK samples, total DNA was extracted from a starting amount of 200 μL of whole blood with the GenElute^TM^ Mammalian Genomic DNA Miniprep Kit (Sigma-Aldrich; www.sigmaaldrich.com) following the manufacturer’s instructions. For the six SUD samples, DNA extraction was performed starting from whole blood preserved on Whatman FTA^TM^ cards (GE Healthcare; www.cytivalifesciences.com) following the manufacturer’s instructions. Finally, for the AMI, MAF, ROM, MOR, EGY and TAN populations, total DNA was obtained from three to five hair bulbs as described in Iamartino et al. [31].

### PCR amplification and sequencing

Polymerase chain reaction (PCR) was used to amplify a 384 base pair (bp) fragment of the mtDNA control (D-loop) region corresponding to nucleotide positions (np) 15386–15769 of the *E*. *asinus* mtDNA reference sequence (Donkey Reference Sequence DRS, GenBank Accession Number NC_001788.1.) [32]. The following primer pair was used: ASS-F 5’-CCCCAAGGACTATCAAGGAAG-3’ [4] and Donk-R 5’-TTGGAGGGATTGCTGATTTC-3’[14]. PCR was carried out in a 25.0 μL reaction volume with 2.0 mM MgCl_2_, 1× PCR Buffer, 0.16 μM for each dNTP, 0.2 μM for each primer, 1 unit of Taq polymerase (AmpliTaq™ DNA Polymerase, Thermo Fisher Scientific; www.thermofisher.com), and 50 ng of DNA. Thermocycling was performed on a GeneAmp* PCR System 9700 thermal cycler (Applied Biosystems; www.thermofisher.com). To avoid possible off-target priming, particularly in the case of low-quality/low-quantity samples as hair bulbs, a touchdown PCR was used to increase the specificity of the amplification process during the first cycles. Detailed cycling conditions are provided in S1 Table. PCR products from each sample were purified prior to sequencing analysis using a Wizard SV Gel and PCR Clean-Up System (Promega; https://ita.promega.com/). Sanger sequencing in the forward direction was performed at the BMR Genomics lab (https://www.bmr-genomics.it/).

### Dataset construction and data analysis

The 374 obtained mtDNA sequences (GenBank Accession Numbers OK431095-OK431468) were compared with 1018 publicly available donkey control-region entries deposited in GenBank (S2 Table). The global dataset included a total of 1392 sequences representing four continents and 67 countries. Regional datasets were also created by aggregating the sequences from the different countries into 13 geographical areas: Eastern Africa (AFE), Northern Africa (AFN), Southern Africa (AFS), Western Africa (AFW), America (AME), Arabian Peninsula (ASA), Central-Western Asia (ASC), Eastern Asia (ASE), Southern Asia (ASS); the Balkans (EUB); Eastern Europe (EUE); Italy (EUI) and Western Europe (EUW) (S2 Table).

Sequences were aligned using CLUSTAL Omega [33] so that vertical columns represented the same nucleotide position for all sequences in the resulting multiple alignment. The multiple alignment was trimmed to the 274 bp overlapping positions available for all sequences (corresponding to nps 15476–15749 of the DRS) and manually edited to remove all insertions/deletions. The number and frequency of the haplotypes, haplotype diversity (*h*) and nucleotide diversity (*n*) [34, 35] were computed with DNASP v.5 [36]. ARLEQUIN 3.5.2.2 package [37] was used to i) perform an Analysis of MOlecular VAriance (AMOVA; [38]) to identify significant partitioning of variation at different hierarchical levels, ii) evaluate past population dynamics through a mismatch distribution analysis, and iii) test the hypothesis of population expansion by calculating Fu’s Fs index [39]. The Harpending’s raggedness index (HRI; [40]) and the sum of squared deviations (SSD) between the observed and expected mismatch were calculated using the method of Schneider and Excoffier [41] implemented in the same package to test the fitting of the observed mismatch distribution curves to a model of sudden population expansion. The demographic expansion parameter τ (time since expansion expressed in units of mutational time) was estimated by a generalized non-linear least-square approach, with confidence intervals computed by parametric bootstrap [41]. Time since expansion (in years) was estimated from τ values through the equation t = τ/2u, where u is the mutation rate per generation [42]. For the calculation, a generation interval of 8 years and a mutation rate of 6.13 x 10^−8^ substitutions per nucleotide/year [2] were used.

Evolutionary relationships between different mtDNA haplotypes were reconstructed using Median-Joining Networks (MJN) with Network v.5.0 software [43]. No priors on the relative weight of mutations occurring at specific positions were applied during the reconstruction of the global network, while the weights of specific positions were changed to reduce the number of cross-links and to increase the discrimination between haplogroups to produce networks from regional datasets (S3 Table). Graphical representations of the networks were obtained with the *in-house* R script CPNetwork (S1 Text) by plotting the regional networks on the network backbone structure derived from the global dataset to better evaluate differences in frequency and geographical distribution of haplotypes.

### MtDNA coding-region: Dataset construction and data analyses

We extended the analysis to the mtDNA coding region to identify diagnostic phylogeny-informative markers that allow dentification of the two previously described clades [4, 11]. A first draft phylogeny was built by performing a preliminary selection of available donkey complete mtDNA sequences from GenBank (S4 Table). MtPhyl v5.003 [44] was used to build a preliminary maximum parsimony (MP) tree based on mtDNA coding-region (i.e., the concatenated sequences of the protein-coding genes) information extracted from complete mitogenomes. The initial tree was carefully inspected to identify potential sequencing problems (gaps) or evolutionary inconsistencies, such as nonsense variants, high numbers of private mutations (>10) and reversions often concentrated in the same region (i.e. in a particular gene). The final preliminary tree accounted for 64 coding-region sequences from 62 domestic donkeys, plus two wild animals (S4 Table). To expand the phylogenetic analysis, we added to this dataset 124 complete mtDNAs extracted and selected from Wang et al. [6] whole-genome sequence (WGS) data as follows: the raw WGS reads of domestic donkeys were downloaded in *fastq* format from Sequence Read Archive (SRA; https://www.ncbi.nlm.nih.gov/sra). The paired-end reads were mapped to the DRS (GenBank accession NC_001788) with bwa v. 0.7.17-r1188 using the *mem* algorithm [45]. Duplicates were removed with samtools v1.9 *rmdup* [46]. Samtools was also used to further filter the alignments keeping only reads mapped in proper pairs and with a mapping quality >30, while removing unmapped reads, secondary and supplementary alignments, and reads failing quality checks. Final BAM files were merged for all those samples with more than one library sequenced using samtools *merge*. A mtDNA consensus sequence was obtained from the mapped reads with ANGSD [47] filtering for a minimum base quality of 20 and a minimum depth of 5 for each base. Positions not fulfilling these criteria were set as ‘N’ and considered as missing positions. Heteroplasmies (i.e., presence of multiple mtDNA variants in the same individual) were called following the International Union of Pure and Applied Chemistry (IUPAC) code (https://genome.ucsc.edu/goldenPath/help/iupac.html) when the less frequent base reached a frequency of 0.15.

Consensus sequences in *fasta* format (restricted to the coding region) were used to build an initial Maximum Parsimony (MP) phylogenetic tree with MtPhyl, based on the previous draft phylogeny, and provisionally renaming Clade 1 as Clade A and Clade 2 as Clade B. We noticed that most of the sequences presented varying numbers of heteroplasmies, ranging from 1 to 201 (0.006–1.3% of the coding region). Notably, all sequences belonging to Clade A had at least one heteroplasmy, and all the samples with the highest numbers belonged to this clade. Since the DRS belongs to Clade B and being aware of the possible presence of NUMTs and the high divergence between the two clades, we decided to realign the reads of samples from Clade A to another sequence (MK982180, #61 in the trees, see S4 Table), used as a reference, which was positioned at the root of Clade A. To compare the results and see if there were differences, we realigned all the sequences to both references and observed the distributions of missing positions and heteroplasmies in the coding region (S1 Fig). The use of a reference internal to each clade seemed to reduce the overall number of missing data (Panels A and C in S1 Fig) and greatly reduced the number of heteroplasmies (Panels B and D in S1 Fig). To test if the differences in the numbers of missing data and heteroplasmies were statistically significant, we applied the R function *test_single_pairs* from Mancuso et al. [48]. The difference in the number of missing data obtained when sequences belonging to Clade A were aligned to either reference was significant (Wilcoxon rank sum test, P<0.001), as was the same difference calculated for Clade B sequences (Wilcoxon rank sum test, P<0.05). Also, the differences in the number of heteroplasmies calculated following the same approach were statistically significant for both Clade A (Wilcoxon rank sum test, P<0.001) and Clade B (Wilcoxon rank sum test, P<0.001).

Given these results and that the realignment to a sequence of the same lineage already proved to be effective in humans [49], we used the alignments to the reference from the same clade for downstream analyses.

Based on the calculated distributions we selected the maximum thresholds for missing data and heteroplasmies and excluded all sequences above these limits. The upper whisker values of the total distribution (S2 Fig) were used as thresholds. We tested the difference in the distributions of missing data and heteroplasmies separately in the two clades when using the respective internal reference. Since the difference in number of missing positions was statistically significant (Wilcoxon rank sum test, P<0.001), two thresholds were used as an exclusion criterion, i.e. five missing positions (0.032% of the coding region) for Clade A and 12 missing positions (0.078% of the coding region) for Clade B (Panel A in S2 Fig and Panel A in S3 Fig). Conversely, the difference in numbers of heteroplasmies was not statistically significant (Wilcoxon rank sum test, P>0.05), so we used five heteroplasmies (0.032% of the coding region) as an exclusion threshold (Panel B in S2 Fig and Panel B in S3 Fig).

After these filtering steps, 100 sequences out of 124 complete mtDNAs were retained (S4 Table), whose average depth of mtDNA coverage ranged from 117X to 2221X.

A maximum parsimony approach and Bayesian computation were applied to infer evolutionary relationships from the final dataset of 164 complete mitogenomes. Only coding-region substitutions were included because of the control-region extraordinary variability and indels’ high instability. MtPhyl was used to build a final MP tree encompassing the total 164 coding-region sequences and including the DRS (S4 Fig). BEAST v2.6.5 [50] was used to generate a Bayesian Skyline Plot (BSP) and a phylogenetic tree rooted using two modern and one ancient horse sequences. Two modern horses (GenBank accessions JN398434 and JN398456) [2] were used as outgroups together with a high coverage mtDNA sequence from an ancient horse (ENA accession SAMEA5408183) [51] radiocarbon-dated to 4.185±0.020 kya. This value was used as a prior together with a previously estimated mtDNA horse mutation rate of 3.25x10^-8^ substitutions per nucleotide/year [2]. The BEAST run was performed under the Hasegawa Kishino Yano (HKY) substitution model with gamma-distributed rates and eight discrete categories, with an uncorrelated relaxed clock model. The ingroup, domestic horses, and clades A, B, B1 and B2 were used as priors and constrained as monophyletic. We ran 20 separate runs, each with a chain length of 10 million iterations with samples drawn every 10000 Markov Chain Monte Carlo (MCMC) steps, after a discarded burn-in of 10% steps. All runs were combined using LogCombiner with default parameters. The maximum clade credibility tree was determined using TreeAnnotator and visualized with R v4.0.4 using the package *ggtree* [52]. The BSP was obtained using Tracer v1.7.1 [53] using a generation time of eight years [6, 7] and plotted in R to explore the trend of effective population size through time.

## Results

### MtDNA coding-region analyses

**Fig 1:**
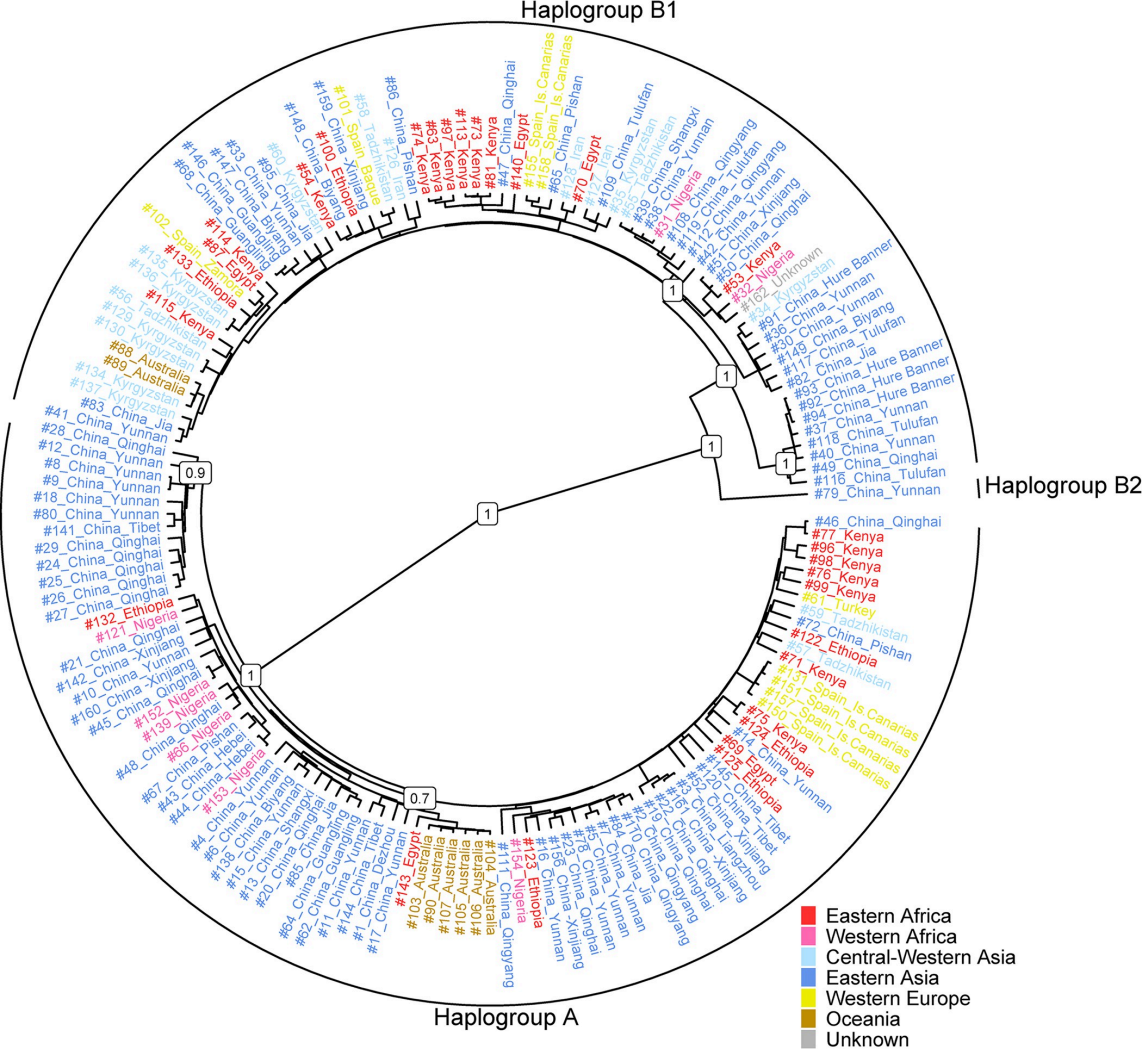
Bayesian phylogenetic tree encompassing 164 donkey coding-region sequences (162 domestic and 2 *Equus africanus somaliensis*). The tree was rooted using two modern and one ancient horse sequences (not shown). Posterior probabilities are indicated for major nodes, including the two major haplogroups. The geographical provenance of the sequences is color-coded as shown in the legend.

The Bayesian and MP tree topologies confirmed the two previously identified major clades of the *E*. *asinus* maternal phylogeny (Fig 1 and S4 Fig): Clade 1 likely derived from the Nubian wild ass (*E*. *a*. *africanus*) and Clade 2 whose origin is still debated [4, 11]. Also, we identified stable coding -region markers that are diagnostic for both clades (S5 Table) and could be used as a basis for a further improvement of the donkey mtDNA phylogeny, especially when exploring potential structure within each macro-haplogroup.

Considering that the number of diagnostic positions differentiating the two clades is comparable to the between-haplogroup mtDNA sequence variation detected in other livestock species [24, 26, 54], here we propose a new nomenclature for the two clades, naming the former Clade 1 (derived from *E*. *a*. *africanus*) as Haplogroup A (HgA) and the former Clade 2 as Haplogroup B (HgB). The latter clade seems to be defined by two sub-branches. One of these, B1, encompasses all the B group sequences, but with the notable exception of a divergent haplotype, that we tentatively named B2 (Fig 1 and S4 Fig).

The age estimates calculated with BEAST are 14.46 kya (95% HPD interval: 8.91–20.98 kya) for clade A, 37.15 kya (95% HPD interval: 17.29–58.79 kya) for clade B, and 19.15 kya (95% HPS interval: 10.79–29.25 kya) for clade B1, when excluding the divergent lineage here named B2 (S5 Fig, panel A). These estimates are much younger than those proposed a few years ago [6, 11]. However, the age of haplogroup B is consistent with the 33ky recently reported by Todd et al. (2022). The demographic trend provided by the BSP (S5 Fig, panel B) shows a stable effective population size until about 20 kya, when a phase of decline began that became steeper from about 15 kya onward, reaching its minimum about 8–10 kya. This phase was followed by a steep increase in size starting around 7 kya, therefore possibly corresponding to the onset of the domestication process.

### MtDNA control-region worldwide dataset

**Fig 2:**
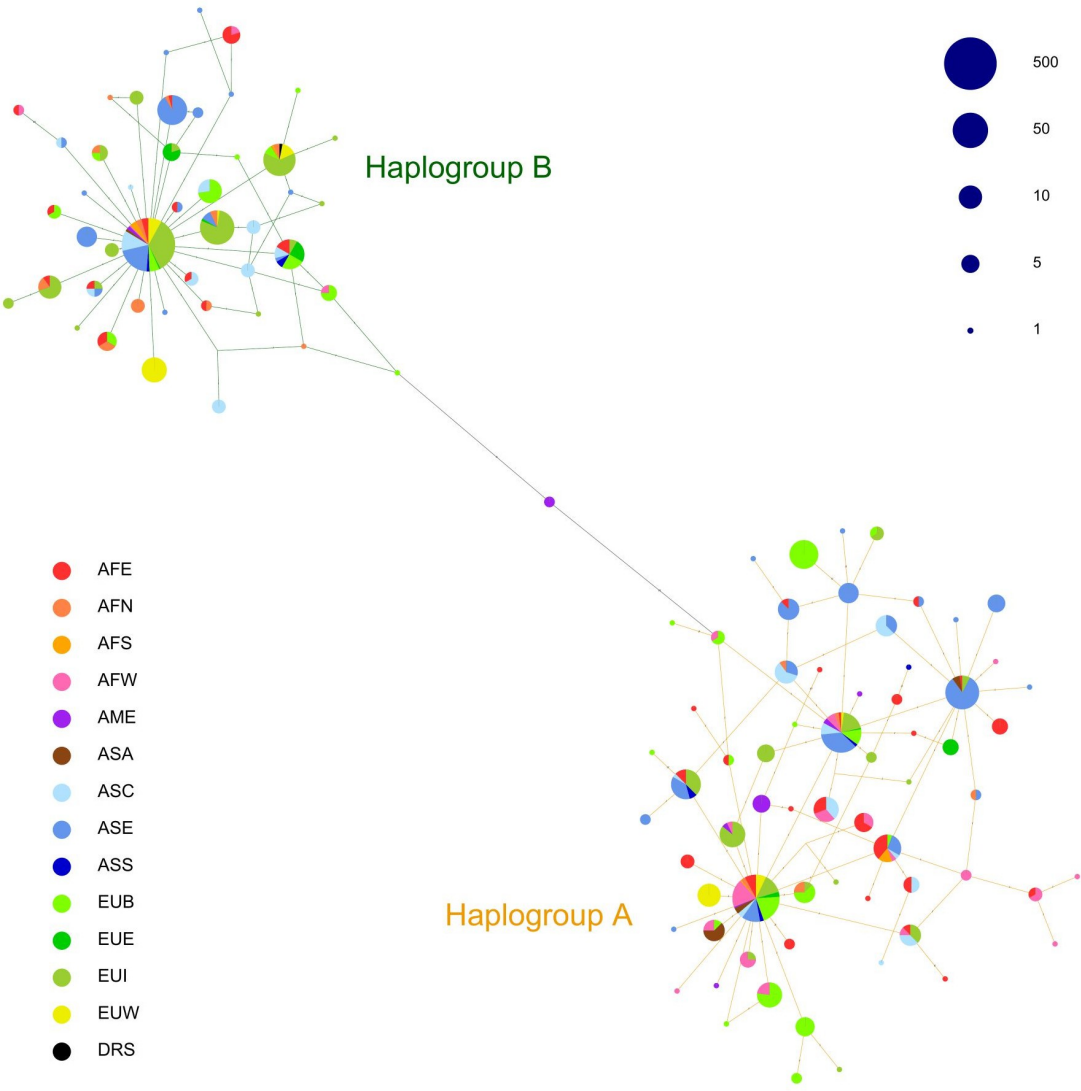
Median-joining network of worldwide donkey control-region variation reconstructed from 1392 mtDNA sequences. The size of the nodes is proportional to haplotype frequencies. Nodes are represented as pie charts indicating the haplotype frequencies in different geographical areas: Eastern Africa (AFE), Northern Africa (AFN), Southern Africa (AFS), Western Africa (AFW), America (AME), Arabian Peninsula (ASA), Central-Western Asia (ASC), Eastern Asia (ASE), Sothern Asia (ASS); Balkans (EUB); Eastern Europe (EUE); Italy (EUI) and Western Europe (EUW). DRS indicates the Donkey Reference Sequence for mtDNA (DRS, NC_001788.1).

The analyzed control-region fragment, corresponding to the hypervariable region I (HVI), showed a remarkable level of sequence variation with 65 variable sites (51 parsimony informative and 14 singletons), 71 substitutions (56 transitions and 15 transversions), and 118 different haplotypes observed over 274 bp and 1392 individuals. Among the 118 different haplotypes, 70 belonged to HgA and 48 to HgB. The most frequent variants were A001 (see S6 Fig for haplotype names and node position on the global network in Fig 2) belonging to HgA (213 sequences, 15% of the total) and B001, belonging to HgB (511 sequences, 37% of the total).

As expected, the control-region median-joining network showed two highly divergent sub-networks (Fig 2) corresponding to the almost equally represented haplogroups A (49.29%) and B (50.71%).

Regional haplogroup distribution showed extensive geographic differentiation (Table 1), with HgA having higher frequencies in Western Africa (92%), Arabian Peninsula (69%), the Balkans (66%) and Eastern Africa (61%) and HgB being more frequent in Southern Africa (81%), Italy (74%), Northern Africa (72%), Western Europe (69%) and Central Asia (66%). America, Eastern Asia, Southern Asia, and Eastern Europe showed a balanced presence of the two clades.

**Table 1:** Summary statistics calculated over the worldwide dataset of donkey mtDNA control-region sequences by geographical region and by country.

Region	Region acronym	Country	Overall				Haplogroup A					Haplogroup B				
			N	nh	*h*	*n*	N	nh	*h*	*n*	%A	N	nh	*h*	*n*	%B
Eastern Africa	AFE	Egypt	21	10	0.800	0.023	8	5	0.786	0.010	38	13	5	0.539	0.003	62
		Eritrea	6	5	0.933	0.030	3	2	0.667	0.010	50	3	3	1.000	0.007	50
		Ethiopia	47	18	0.911	0.022	33	14	0.850	0.007	70	14	4	0.692	0.004	30
		Kenya	5	4	0.900	0.026	3	3	1.000	0.005	60	2	1	⎯	⎯	40
		Somalia	6	6	1.000	0.033	3	3	1.000	0.019	50	3	3	1.000	0.007	50
		Sudan	18	14	0.967	0.023	13	10	0.949	0.010	72	5	4	0.900	0.008	28
		AFE overall	103	34	0.922	0.025	63	22	0.916	0.010	61	40	12	0.687	0.005	39
Northern Africa	AFN	Algeria	5	4	0.900	0.028	3	2	0.667	0.005	60	2	2	1.000	0.004	40
		Libya	8	3	0.607	0.012	1	1	1.000	⎯	12	7	2	0.476	0.002	88
		Morocco	31	11	0.824	0.019	9	4	0.694	0.004	29	22	7	0.693	0.004	71
		Tunisia	6	4	0.800	0.016	1	1	1.000	⎯	17	5	3	0.700	0.003	83
		AFN overall	50	15	0.802	0.019	14	5	0.725	0.004	28	36	10	0.654	0.004	72
Southern Africa	AFS	South Africa	5	3	0.700	0.020	4	2	0.500	0.004	80	1	1	1.000	⎯	20
		Swaziland	5	1	⎯	⎯	⎯	⎯	⎯	⎯	⎯	5	1	⎯	⎯	100
		Tanzania	2	2	1.000	0.040	1	1	1.000	⎯	50	1	1	1.000	⎯	50
		Zambia	3	1	⎯	⎯	⎯	⎯	⎯	⎯	⎯	3	1	⎯	⎯	100
		Zimbabwe	11	2	0.182	0.001	⎯	⎯	⎯	⎯	⎯	11	2	0.182	0.001	100
		AFS overall	26	4	0.403	0.015	5	2	0.600	0.004	19	21	2	0.095	0.000	81
Western Africa	AFW	Benin	4	4	1.000	0.024	3	3	1.000	0.007	75	1	1	1.000	⎯	25
		Burkina Faso	46	13	0.627	0.007	44	11	0.592	0.004	96	2	2	1.000	0.007	4
		Chad	5	4	0.900	0.019	4	3	0.833	0.005	80	1	1	1.000	⎯	40
		Ghana	3	2	0.667	0.005	3	2	0.667	0.005	100	⎯	⎯	⎯	⎯	⎯
		Guinea	5	3	0.800	0.004	5	3	0.800	0.004	100	⎯	⎯	⎯	⎯	⎯
		Mali	5	4	0.900	0.004	5	4	0.900	0.004	100	⎯	⎯	⎯	⎯	⎯
		Mauritania	4	3	0.833	0.027	3	2	0.667	0.005	75	1	1	1.000	⎯	25
		Niger	6	5	0.933	0.030	4	3	0.833	0.016	67	2	2	1.000	0.015	33
		Senegal	6	3	0.600	0.002	6	3	0.600	0.002	100	⎯	⎯	⎯	⎯	⎯
		AFW overall	84	22	0.752	0.011	77	18	0.706	0.006	92	7	4	0.714	0.008	8
Americas	AME	Mexico	27	9	0.795	0.021	14	7	0.824	0.006	52	13	2	0.282	0.006	48
		AME overall	27	9	0.795	0.021	14	7	0.824	0.006	52	13	2	0.282	0.006	48
Arabian Peninsula	ASA	Oman	5	3	0.800	0.006	5	3	0.800	0.006	100	⎯	⎯	⎯	⎯	⎯
		Saudi Arabia	5	3	0.800	0.026	3	2	0.667	0.005	60	2	1	⎯	⎯	40
		United Arab Emirates	6	3	0.733	0.026	3	2	0.667	0.002	50	3	1	⎯	⎯	50
		Yemen	10	4	0.733	0.021	7	2	0.476	0.002	70	3	2	0.667	0.002	30
		ASA overall	26	13	0.757	0.020	18	9	0.621	0.004	69	8	4	0.250	0.001	31
Cental-West Asia	ASC	Azerbaijan	3	2	0.667	0.024	1	1	1.000	⎯	66	2	1	⎯	⎯	33
		Iran	7	4	0.857	0.022	2	1	⎯	⎯	29	5	3	0.800	0.004	71
		Iraq	5	2	0.600	0.020	2	1	⎯	⎯	40	3	1	⎯	⎯	60
		Israel	5	2	0.600	0.026	3	1	⎯	⎯	60	2	1	⎯	⎯	40
		Jordan	5	4	0.900	0.029	2	1	⎯	⎯	40	3	3	1.000	0.005	60
		Kyrgyzstan	3	3	1.000	0.005	⎯	⎯	⎯	⎯	⎯	3	3	1.000	0.005	100
		Lebanon	5	2	0.600	0.026	2	1	⎯	⎯	40	3	1	⎯	⎯	60
		Syria	5	3	0.800	0.020	2	1	⎯	⎯	40	3	2	0.667	0.002	60
		Turkey	82	14	0.694	0.019	27	8	0.829	0.006	33	55	6	0.357	0.002	67
		Turkmenistan	2	1	⎯	⎯	⎯	⎯	⎯	⎯	⎯	2	1	⎯	⎯	100
		Uzbekistan	3	2	0.667	0.027	1	1	1.000	⎯	33	2	1	⎯	⎯	67
		ASC overall	125	39	0.763	0.020	42	16	0.871	0.007	34	83	23	0.492	0.003	66
Eastern Asia	ASE	China	287	37	0.846	0.022	142	20	0.861	0.006	49	145	17	0.529	0.003	51
		Mongolia	3	2	0.667	0.027	1	1	1.000	⎯	33	2	1	⎯	⎯	67
		Vietnam	3	2	0.667	0.027	1	1	1.000	⎯	33	2	1	⎯	⎯	67
		ASE overall	293	37	0.842	0.022	144	20	0.861	0.006	49	149	17	0.518	0.003	51
Southern Asia	ASS	Bangladesh	4	2	0.500	0.018	1	1	1.000	⎯	25	3	1	⎯	⎯	75
		India	10	4	0.644	0.019	3	2	0.667	0.002	30	7	2	0.286	0.001	70
		Nepal	4	4	1.000	0.021	3	3	1.000	0.005	75	1	1	1.000	⎯	25
		Pakistan	4	2	0.500	0.009	4	2	0.500	0.009	100	⎯	⎯	⎯	⎯	⎯
		ASS overall	22	6	0.766	0.022	11	4	0.691	0.005	50	11	2	0.327	0.001	50
Balkan Europe	EUB	Albania	19	11	0.901	0.022	11	6	0.800	0.006	58	8	5	0.786	0.005	42
		Bulgaria	22	10	0.844	0.020	14	6	0.681	0.005	64	8	4	0.750	0.005	36
		Croatia	51	13	0.794	0.020	34	7	0.601	0.007	67	17	6	0.713	0.004	33
		Greece	23	7	0.771	0.019	8	4	0.750	0.003	35	15	3	0.514	0.002	65
		Kosovo	10	4	0.800	0.016	8	3	0.714	0.003	80	2	1	⎯	⎯	20
		Macedonia	13	4	0.654	0.008	12	3	0.591	0.002	92	1	1	1.000	⎯	8
		Montenegro	14	9	0.901	0.022	8	6	0.893	0.005	57	6	3	0.600	0.002	43
		Romania	10	3	0.622	0.003	10	3	0.622	0.003	100	⎯	⎯	⎯	⎯	⎯
		Serbia	10	3	0.711	0.015	8	2	0.571	0.002	80	2	1	⎯	⎯	20
		EUB overall	172	31	0.876	0.019	113	19	0.783	0.006	66	59	12	0.732	0.004	34
Eastern Europe	EUE	Hungary	3	3	1.000	0.027	1	1	1.000	⎯	33	2	2	1.000	0.007	67
		Poland	5	2	0.600	0.022	2	1	⎯	⎯	40	3	1	⎯	⎯	60
		Russia	10	3	0.711	0.023	4	1	⎯	⎯	40	6	2	0.533	0.002	60
		Ukraine	10	2	0.533	0.019	6	1	⎯	⎯	60	4	1	⎯	⎯	40
		EUE overall	28	7	0.839	0.022	13	3	0.564	0.005	46	15	4	0.743	0.004	54
Europe Italy	EUI	Italy	347	28	0.697	0.017	90	14	0.816	0.005	26	257	14	0.469	0.002	74
		EUI overall	347	28	0.697	0.017	90	14	0.816	0.005	26	257	14	0.469	0.002	74
Western Europe	EUW	France	5	2	0.600	0.024	2	1	⎯	⎯	40	3	1	⎯	⎯	60
		Portugal	6	3	0.600	0.015	1	1	1.000	⎯	17	5	2	0.400	0.001	83
		Spain	77	6	0.736	0.018	25	3	0.587	0.002	32	52	3	0.511	0.002	68
		United Kingdom	1	1	1.000	⎯	⎯	⎯	⎯	⎯	⎯	1	1	1.000	⎯	100
		EUW overall	89	7	0.725	0.018	28	3	0.561	0.002	31	61	4	0.501	0.002	69
Worldwide			1392	227	0.833	0.022	632	130	0.854	0.007	45	760	97	0.540	0.003	55

N = total number of sequences; nh = number of haplotypes; h = haplotype diversity; n = nucleotide diversity; %A = percentage of sequences belonging to Haplogroup A; %B = percentage of sequences belonging to Haplogroup B.

Haplotype diversity (Table 1) showed the highest value in Eastern Africa (*h* = 0.922) followed by Eastern Europe (*h* = 0.839), while the lowest value was found in Southern Africa (*h* = 0.403). Eastern Africa also showed the highest nucleotide diversity (*n* = 0.025, Table 1) followed by Eastern Asia, Southern Asia, and Eastern Europe (all with *n* = 0.022). At the haplogroup level, both haplotype and nucleotide diversity were higher within HgA (*h* = 0.854, *n* = 0.007) compared to HgB (*h* = 0.540, *n* = 0.003), showing the highest values for HgA in AFE (*h* = 0.916), where Ethiopia and Sudan possessed a very high numbers of HgA haplotypes (nh = 14 and nh = 10, respectively; Table 1), and in the EUE (*h* = 0.743) for HgB, followed by EUB, AFE and AFW (Table 1).

According to the results of AMOVA, 22.25% (Table a in S6 Table) and 14.43% (Table b in S6 Table) of variance explained differentiation between regions and countries, respectively. More than 88% of the mtDNA variation was distributed between the two haplogroups, while only a small fraction was found between regions (1.17%, Table c in S6 Table) and countries (2.18%, Table d in S6 Table) within haplogroups. When AMOVA was performed using HgA and HgB subsets separately, the differentiation between regions and countries respectively accounted for 10.89% (Table e in S6 Table) and 19.84% (Table g in S6 Table) for HgA, and for 7.55% (Table f in S6 Table) and 13.92%, (Table h in S6 Table) for HgB.

The overall mismatch distribution showed a bimodal curve (Top left panel in S7 Fig). The first peak with a maximum of 1 pairwise difference corresponded to the mismatches between haplotypes belonging to the same haplogroup. The second peak with maximum at 11 pairwise differences corresponded to the mismatches between haplotypes from the two haplogroups.

Within-clade mismatch distribution analysis revealed a unimodal distribution for HgA with individuals differing mostly by 1–2 mismatches (Top central panel in S7 Fig), while for HgB the curve showed a half-bell shape with modal value at 0 pairwise differences (Top right panel in S7 Fig), indicating overall a more recent demographic expansion compared to HgA.

Mismatch distribution analyses were carried out also separately for the different geographical areas (S7 Fig). Due to the occurrence of both clades in every area, all the overall plots showed the expected bimodal behavior, while within-clade analyses returned unimodal distributions. Ragged curves were observed when the number of sequences per clade was below ca. 20, as in the case of AFS, AME, ASS and EUE and of HgB in AFW and ASA. Due to this behavior, all these cases were excluded from further analyses. For HgA, AFE mismatch distribution showed a bell-shaped curve with a peak at 3 mismatches, followed by ASC with a peak at 2 mismatches, while all other regions had peaks at 1 mismatch except for ASA with a peak at 0. For HgB, the AFE, AFN and EUB showed peaks at 1 mismatch, while the trend for all the remaining regions was that of a half-bell curve with a peak at 0 mismatches.

All values of Fu’s Fs index being negative, large, and statistically significant (S7 Table) indicated a population expansion at the regional level, except for the global dataset including all HgA and HgB sequences, which had a non-statistically significant value of Fs = 0.000 (P = 0.578). Statistical significance for either Harpending’s raggedness index (HRI) or Sum of Squared Deviations (SSD) values (S7 Table) for ALL, AFW, ASE, EUI and EUW regions within HgA indicated that the respective mismatch distributions did not fit a model of sudden population expansion.

The calculation of expansion times based on τ (S8 Table) provided values that were higher for HgA compared to HgB both on the global and the regional datasets, with the only exception of ASE which showed the opposite behavior. Overall, HgA and HgB expansion times were dated to 7.154 kya and 3.267 kya, respectively. Within HgA, AFE scored the oldest expansion time, i.e. 11.024 kya, followed by ASC (8.341 kya) and ASE (7.024 kya), while the most recent expansion times were those of EUW (3.115 kya) and AFW (1.751 kya). For HgB the oldest expansion times were calculated for ASE (8.470 kya), EUB (4.791 kya) and AFN (3.980 kya), while the most recent for EUW (2.723 kya) and EUI (2.472 kya).

The median-joining (MJ) network revealed the occurrence of star-like structures in both haplogroups (Fig 2), a pattern typical of populations characterized by demographic expansion. Anyway, variation seemed to be structured differently within the two clades: HgA showed two highly represented core haplotypes (A001 and A002, accounting for 34% and 15% of HgA sequences, respectively), and a high number of minor haplotypes connected to the central ones and to each other by several cross-links and separated by 1 to 5 mutational steps. HgB had a single central haplotype (B001, representing 67% of HgB sequences) with most of the minor ones differing from the core by a single mutational step and stemming from it on separate branches with only a few cross-links.

The major haplotypes of the two haplogroups A001 (HgA) and B001 (HgB) were separated by 11 mutations, corresponding to positions 15654, 15590, 15579, 15599, 15645, 15699, 15484, 15490, 15503, 15667, 15625 of the DRS. HgA A001 and A002 haplotypes were present in all regions, with the exception of A002 lacking in AFS and ASA (S8 Fig). HgB B001 was found in all regions except AFS. Some haplotypic variants were private to specific geographical regions (S8 Fig).

Despite the different numbers of sequences available per geographical area (Table 1), the regional networks (S8 Fig) provided interesting evidence on the structuring of variation worldwide. Eastern Africa (AFE), the likely domestication center, displayed the highest number of haplotypes overall, but the distribution of HgA and HgB variants was remarkably different between the other African regions: Western Africa (AFW) showed a predominance of HgA sequences, accompanied by a few distantly related HgB sequences, opposite to Northern Africa (AFN) where HgB sequences occurred more frequently, with only a minor presence of a few HgA haplotypes separated by several mutational steps.

**Fig 3:**
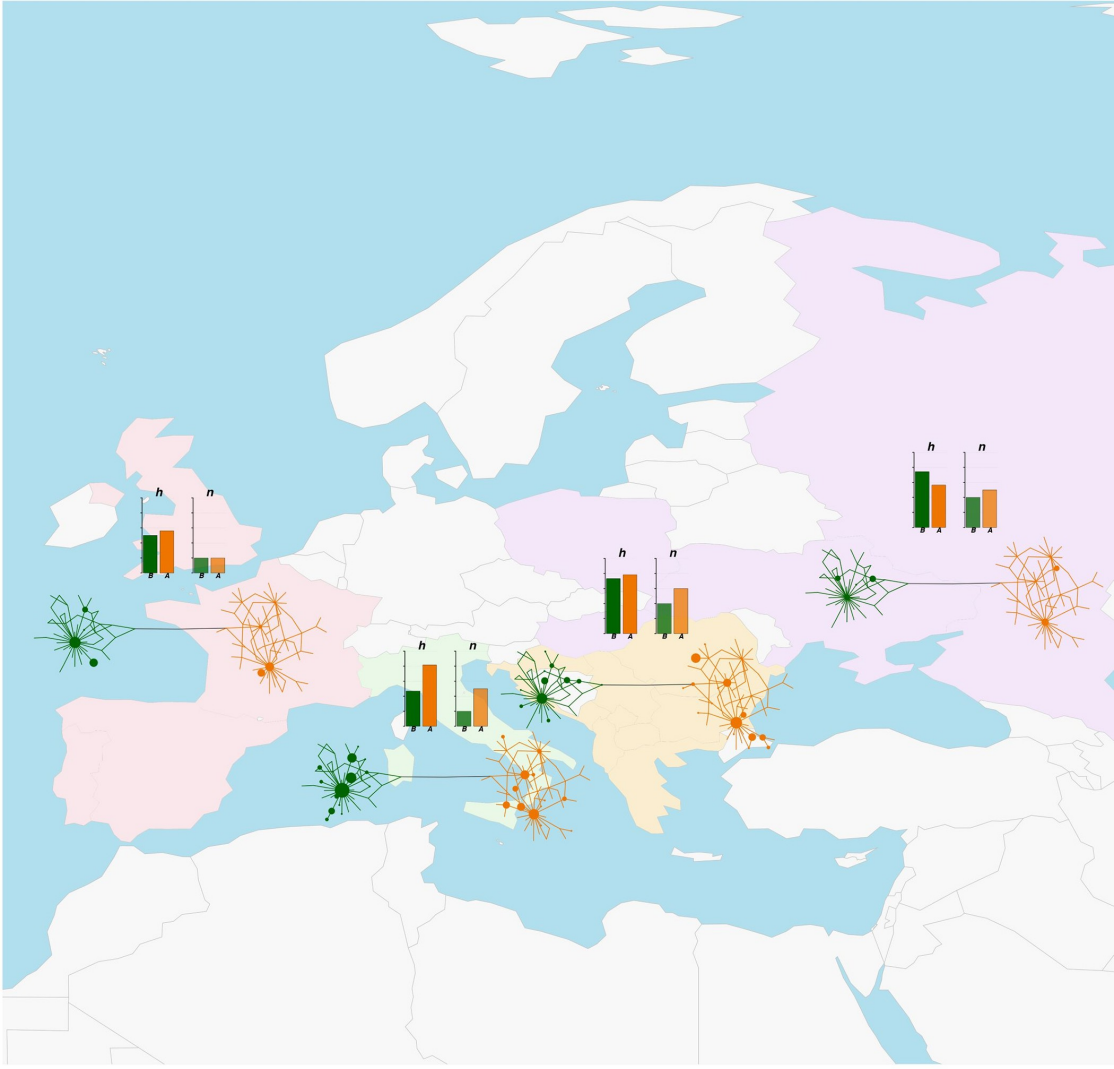
Map of regional Median-joining networks, haplotype and nucleotide diversity values for Europe. Continental map of Europe showing regional Median-joining networks and histograms of haplotype (*h*, darker shade) and nucleotide (*n*, lighter shade) diversity values for haplogroups A (orange shades) and B (green shades). The map was plotted with the *getMap* and *mapCountryData* functions of the R package *rworldmap* [55]. The labelling of geographical areas is the same as in Fig 2. A detailed representation of regional Median-joining networks is given in S8 Fig.

**Fig 4:**
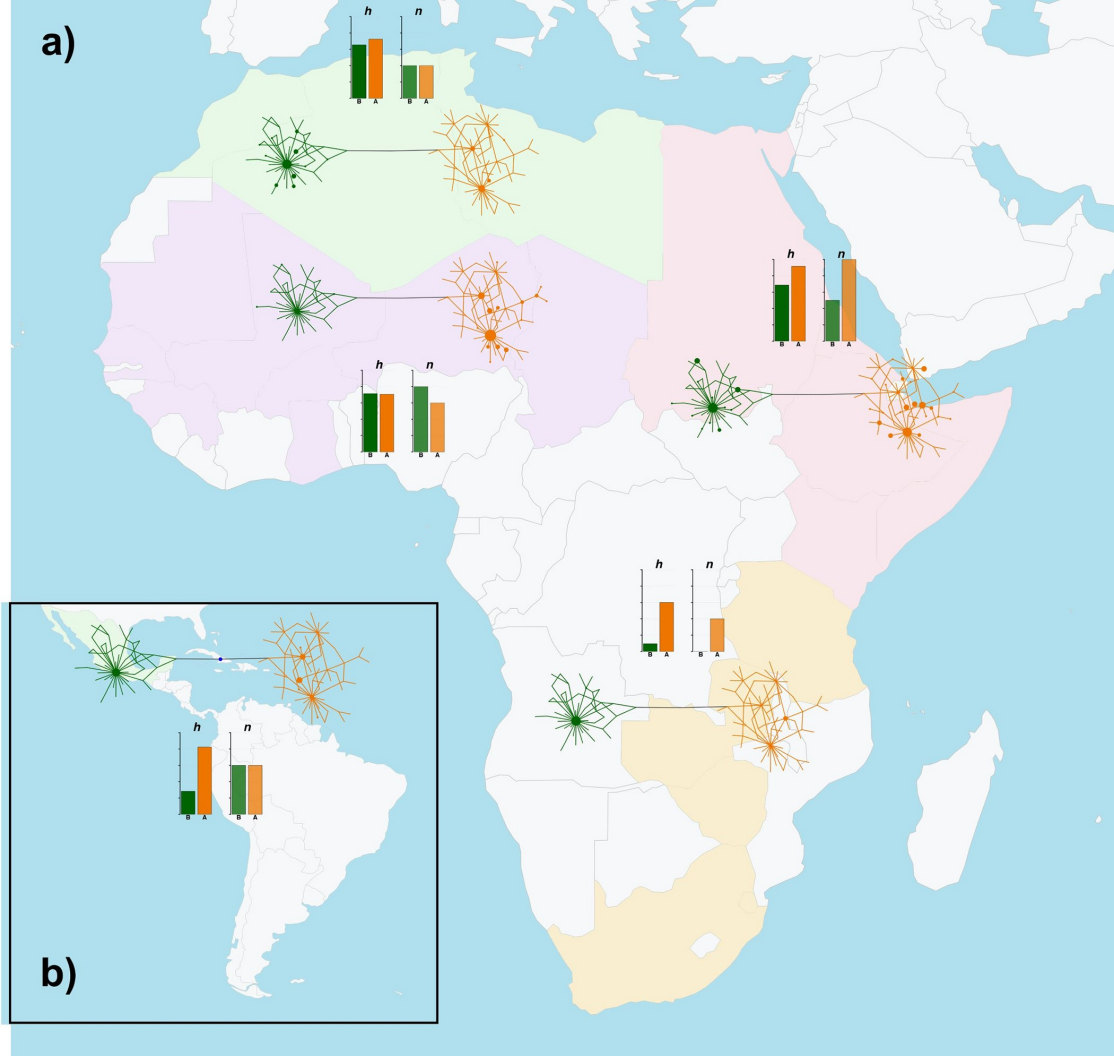
Map of regional Median-joining networks, haplotype and nucleotide diversity values for Africa and Central America. Continental maps of Africa (panel **a**) and Central America (panel **b**) showing regional Median-joining networks and histograms of corresponding haplotype (*h*, darker shade) and nucleotide (*n*, lighter shade) diversity values for haplogroups A (orange shades) and B (green shades). The map was plotted with the *getMap* and *mapCountryData* functions of the R package *rworldmap* [55]. The labelling of geographical areas is the same as in Fig 2. A detailed representation of regional Median-joining networks is given in S8 Fig.

**Fig 5:**
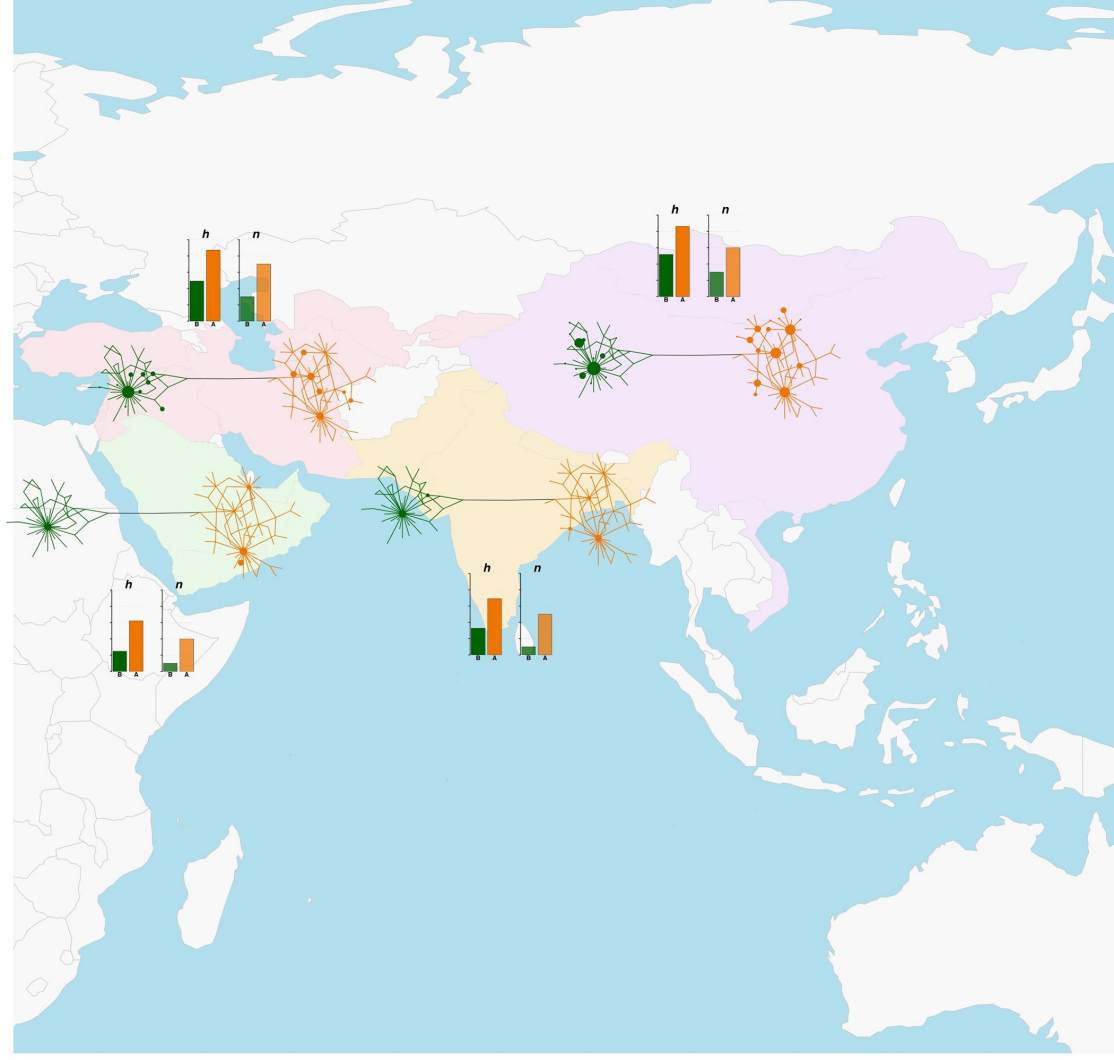
Map of regional Median-joining networks, haplotype and nucleotide diversity values for Asia. Continental map of Asia showing regional Median-joining networks and histograms of corresponding haplotype (*h*, darker shade) and nucleotide (*n*, lighter shade) diversity values for haplogroups A (orange shades) and B (green shades). The map was plotted with the *getMap* and *mapCountryData* functions of the R package *rworldmap* [55]. The labelling of geographical areas is the same as in Fig 2. A detailed representation of the regional Median-joining networks is given in S8 Fig.

The geographical areas for which a high number of sequences were available, namely Central-Western Asia (ASW), Eastern Asia (ASE), Balkan Europe (EUB) and Italy (EUI), were all highlighted as diversity-rich regions by our analyses. For areas with a lower number of sequences, instead, separating actual diversity levels from sampling effects was not straightforward. Nevertheless, if we consider that Southern Africa (AFS), Arabian Peninsula (ASA), Americas (AME), Southern Asia (ASS) and Eastern Europe (EUE) displayed a similar number of sequences (n = 22–28, Table 1), these areas showed clear differences in the distribution of variation and in the number of detected haplotypes (Figs 3–5 and S8 Fig). ASA and AFS, in particular, had a lower number of haplotypes compared to EUE and AME (Table 1). Together with a relatively high number of haplotypes, this last area also showed the presence of the only haplotype variant intermediate between HgA and HgB and an uneven occurrence of HgB variants with respect to the predominant HgA haplotypes, similar to the pattern found in AFW (Fig 4).

## Discussion

Due to its minor role in present-day agriculture and transportations, particularly in industrialized countries, and the lack of large-scale economic interest, the domestic donkey has been neglected also from a molecular perspective compared to other livestock species. A first reference genome sequence has become available only a few years ago [56] and donkey-specific single nucleotide polymorphism (SNP) panels are not available yet. In line with this general trend, also the nomenclature of donkey mtDNA haplogroups has been overlooked.

The analysis of complete mtDNA sequences we carried out led to the identification of lineage-diagnostic coding-region mutational motifs. Based on this evidence, we propose a new nomenclature for the two mtDNA clades, in which the former Clade 1 and Clade 2 are renamed as Haplogroup A (HgA) and Haplogroup B (HgB), respectively. Within HgB, the identification of a deep-diverging haplotype might represent a previously unknown sub-lineage that left a marginal trace (Fig 1 and S5 Fig). The possibility that this haplotype might represent a novel and divergent sub-haplogroup B2 needs be confirmed and further described by extending the number of analyzed sequences. Low-frequency highly divergent mtDNA lineages have been described in several livestock species [24, 26, 57, 58] and the introgression from divergent evolutionary lineages has recently been detected in both modern and ancient donkey genomes from various regions of the world, pointing to sporadic but detectable genomic contributions either from Asian kiangs or other sources [7].

Although the sampled donkey mitogenomes are far from representing a population under panmixia, the pre- and post-domestication demographic trends depicted by the BSP provided a good visualization of effective population size changes over time (S5 Fig, panel B), in line with previous estimates [6, 7]. The trend highlighted in donkeys matches those described in livestock species in general, in which demographic trajectories show the overlapping effects of the Pleistocenic glacial events and of the domestication process [26, 54, 59].

The analysis of 1392 control-region sequences encompassing 118 different haplotypes from Africa, Asia, Central America, and Europe, allowed us to draw a comprehensive overview of matrilineal genetic diversity and evolutionary relationships among donkey populations worldwide, confirming the effectiveness of mtDNA for the assessment of variation patterns over broad geographical areas.

At a shallow scale our results confirmed the major outcomes of previous donkeys mtDNA studies [4, 11]: the occurrence of the two haplogroups, HgA and HgB (Fig 2), differing in terms of levels of variation and structuring of diversity, with HgB showing in general lower values of both haplotype and nucleotide diversity (Table 1). The structure of Median-joining network and the within-clade mismatch distribution curves accounted for a higher structuring of variation for HgA relative to HgB (Fig 2 and S7 Fig). The estimated expansion times (S8 Table) confirmed an earlier demographic increase for HgA dating back to 7.154 kya, which fits well with the date of donkey domestication (ca. 7500–6500 years ago) suggested on the basis of non-molecular evidence [9]. Conversely, the more recent expansion of HgB was dated to 3.267 kya (Fig 2 and S7 Fig), which is in agreement with the results of Beja-Pereira et al. [4], who suggested a likely dual domestication from highly diverged wild populations for HgA and B sequences, and with those of Kimura et al. [11] showing that HgA topology is coherent with a scenario in which the Nubian wild ass was domesticated in several areas over an extended period, with multiple introgression events from wild populations, and different from the less complex domestication process starting from fewer founders from another ancestral population for HgB. In contrast to this shared view, a recent investigation based on whole-genome sequences of both modern and ancient donkeys suggested that, considering the similar Time to Most Recent Common Ancestor (TMRCA) values of ca. 39–32 kya found for both HgA and HgB, the two lineages could have coexisted in sympatry at the time when donkeys were first domesticated and speculated on a single and early domestication event in Africa followed by a spread at an even rate into the Arabian Peninsula and Eurasia [7]. In this scenario, the differences we scored in terms of expansion times for the two haplogroups overall and at the regional level can be accounted for by several factors acting globally (e.g. different evolutionary dynamics for HgA and HgB lineages leading to a higher/lower within-lineage diversity preceding domestication) and/or locally (e.g. unbalanced amount of initial variation due to random sampling and founder effect, different demographic trajectories and/or repeated introgression from wild asses of lineage A in Eastern Africa and surrounding areas). Unfortunately, the geographical and temporal distributions of donkey ancient DNA data are too ragged to fully substantiate either hypothesis, with a dramatic lack of molecular evidence from the putative domestication center dating back to the pre- and early post-domestication times.

As successfully demonstrated by other studies on livestock domestication and post-domestication history [60–63], the analysis of molecular variation within a geographical framework is useful to better highlight the relationships between populations from different areas of the world. The analyses we performed at the regional level highlighted interesting differences between geographical areas (Figs 3–5 and Table 1). Overall, a geographical structuring of diversity, particularly within haplogroups, was highlighted by AMOVA results, which is in line with evidence from WGS data showing a major split between donkey populations from different continents and a further within-continent sub-structuring [6, 7]. For HgA, Africa, Asia and Europe all presented regions with a highly structured variation, while present-day HgB diversity seemed to be centered around the Mediterranean basin (Figs 3–5).

Our evidence further support the view that either domestication occurred in Eastern Africa [4, 5] or that donkeys domesticated elsewhere in Africa entered this region more recently [7]. In fact, AFE showed the highest values of diversity metrics overall (haplotype and nucleotide diversities; Fig 4 and Table 1), and signs of the earliest population expansion (S7 Fig and S7, S8 Tables), confirming the findings of mtDNA- and WGS-based studies indicating that the modern donkeys from the Horn of Africa and Kenya so far best represent the descendants of earliest domesticates [4, 5, 7].

The evidence derived from the within-Clade mismatch distribution and expansion time calculations by region overall indicated an earlier expansion for HgA compared to HgB. Within HgA, following AFE, the signals of older population expansion were respectively found in ASC, ASE, EUB and EUI, while other regions within or close to Africa like AFN, ASA and AFW seemed to have had populations that expanded later (S8 Table). Within HgB, among the African regions the oldest expansion time was found for the AFN population, but it was predated by ASC, ASE and EUB expansion times. This latter evidence is apparently at odds with the shared view of an African origin of the wild ancestor of HgB domestic donkeys. Even if the calculation of expansion times may have been affected to some extent by the higher number of sequences available in ASC, ASE and EUB compared to AFN, under the hypothesis of a dual domestication, it can be speculated that after domestication the diffusion and population expansion of the early HgB domesticates may have occurred more effectively in Asia and South-Eastern Europe than within the African continent due to the presence of the Sahara acting as a barrier. Indeed, donkeys are not found in the archaeological series of AFW until the beginning of the common era, which may indicate an early yet undocumented arrival in the Western African continent or a slow westward migration, only reaching the modern range later [7].

Particularly striking was the strongly uneven distribution of HgA and HgB sequences between African regions (Fig 4). Except for Eastern Africa, in fact, all other areas displayed a remarkable unbalance in the occurrence of HgA and HgB variants. In particular, we observed an opposite pattern when comparing AFN *vs* AFW and AFS which showed a respective prevalence of HgA (77 out of 84 total sequences, 92%) and HgB sequences (36 out of 50, 72% in AFW; 21 out of 26, 81% in AFS). This pattern confirms the evidence described by Ma et al. [12] and could be explained by a strongly limited exchange of animals between African regions due to the presence of the Sahara.

ASE and ASC, both characterized by a large sample size, showed the presence of several HgA private haplotypes related to each other that do not occur elsewhere in the world, which fits well with the remarkable population expansion that HgA experienced within these areas (S8 Table).

The network of Central America (AME) may have been strongly affected by sampling bias due to the low number of sequences and haplotypes, thus it was not possible to draw precise conclusions regarding the relationships between Central American animals and the donkey populations from other areas. Nevertheless, the sharing of some haplotypes between AME, AFW and AFN (Figs 3, 4, and S8 Fig) is in line with evidence of North-Western African contributions to Central and Southern American livestock genetic makeup [62, 64], besides the known introductions from the Iberian Peninsula starting from the 15^th^ Century of the Common Era (CE) [65].

The uneven distribution of HgA and HgB diversity between geographical regions, together with the regional within-clade shapes of mismatch distribution curves and population expansion times, suggest that different temporal and spatial dynamics may have shaped the distribution of donkey mtDNA variation around the world.

The hypothesis of non-synchronous diffusion and expansion of HgA and HgB lineages has already been postulated based on previous research by Stanisic et al. [21] and Ma et al. [12] for the Balkans and China, respectively. Based on our evidence, this scenario may apply also to other areas, since in general HgA diffusion seemed to have preceded that of HgB by approximately at least one thousand years in most regions (S8 Table). HgA evidence matches the scenario of donkey domestication carried out by Eastern African herders around 7.5–6.5 kya in response to the increasing desertification of the Sahara and climatic deterioration of eastern Africa [8, 9]. Assuming a more recent time frame for HgB domestication and subsequent expansion, it likely occurred when the aridification of Sahara had already become extensive, and thus the desert may have acted as a barrier to the diffusion of HgB lineage to the west and south of the continent.

Contrary to the expectations based on previous research [66] and on its geographical proximity to the putative domestication center, the Arabian Peninsula did not show particularly high diversity estimates (Table 1). On the contrary, ASE, EUB, and EUI (Figs 3–5 and Table 1) were all highlighted as regions harboring high levels of diversity with populations bearing signs of remarkable expansions for both haplogroups. This outcome is not surprising considering that all these areas represented crossroads for migrations and trades since pre-historical times and that donkeys have played a role in the transportation of people and goods, particularly as burden animals, along continental routes for about five millennia [17, 66, 67]. Continental Asia, in particular, has been crossed by land trade routes like the famous Silk Road with clues of east-west cultural and material exchanges as early as 3.5 kya [68]. The remarkable diversity we observed in ASE can be accounted for by multiple ancient introductions of donkeys in this region followed by a fast demographic expansion. Han et al. [17] indicated that, although rare, donkeys were already present in China 4 kya, and that from about 2 kya these animals spread widely into the west and north of the country. Also, the gradients of mtDNAdiversity found by Ma et al. [12] suggested that animals belonging to the two lineages may have been introduced into China along different migration routes.

The Balkans, besides representing the contact zone between eastern and western cultures, are also known to have acted as the entry point of livestock species into Europe by terrestrial roads [21, 69]. Thus, the high mtDNA diversity recorded in this area, particularly for HgB, may derive from historical extensive exchanges of animals between Central-Eastern Europe and South-Western Asia, combined with the mixed unplanned mating adopted by donkey breeders in the Balkan region [21].

Similarly, the Italian peninsula, due to its position in the middle of the Mediterranean basin, has had a long history of commerce, invasions, and migrations, which led to the high levels of livestock diversity already found in several species [62, 70].

Although based on a small sample set, the occurrence of shared haplotypic variants between AFW and AME, and between AFN and EUW pointed at known and well documented migration and trade events. Extensive gene flow between Northern African and Western European populations, in fact, has been detected in several livestock species [62, 71] including donkey [7, 12, 65], as well as between Western Africa and Central and Southern America along the Atlantic Ocean trade route [7, 62, 64]. Furthermore, the AME network (Fig 4) showed a unique feature, i.e. the occurrence of a haplotype (B029) with a position and a mutational motif intermediate between those of HgA and HgB. This haplotype was found in two Mexican Creole donkeys [15] and has not been recorded elsewhere in the world, so far. At least three hypotheses can be proposed to explain this occurrence: (i) the sequences represent laboratory artefacts deriving from an undetected cross-contamination between HgA and HgB samples; (ii) back mutations have affected by chance exactly some of the clade-diagnostic positions (either 15484, 15490, and 15503 or 15599, 15645, and 15669), or more intriguingly (iii) this variant is the only known remnant of an additional mitochondrial haplogroup belonging to an unknown ancestral population with a mutational motif intermediate between HgA and B.

Carrying out a comprehensive geographical-based analysis of mtDNA variation is influenced by the availability of region-specific information and by the evenness of data density across the study area. In the case of the present study, we tried to minimize the effects of sample size differences among geographical areas by joining information from multiple studies. However, many regions of the African continent were underrepresented (e.g. South-Western Africa) and there was a remarkable lack of publicly available information for the Americas. Data availability and level of variation may also have been affected by factors as ongoing or recent conservation programs or breeding plans carried out locally, which, on one side, may stir the production of molecular data for local breeds, while, on the other side, may affect the levels of variation scored in some populations e.g. by constraining gene flow or increasing diversity through crossbreeding, thus biasing the geographical distribution of mtDNA variants. As for the phylogenetic analyses, a possible limitation of this study is the identification of the deep-diverging B2 lineage based on a single haplotype (although with stable diagnostic markers, S4 Fig), which should be further investigated increasing the number of complete mtDNAs, and possibly adding ancient DNA data.

Nevertheless, the global picture emerging from our analyses points at the occurrence of high underlying mtDNA variation worldwide, accompanied by moderate levels of geographical structuring between areas and haplogroups, despite the significant contraction of donkey census sizes in many countries. This knowledge, together with the availability of whole-genome sequences for both modern and ancient donkeys from many regions of the world could be exploited to devise new cost-effective tools allowing to enable an extensive characterization of nuclear genetic variability. These tools, like the commercial SNP arrays already available for all the major livestock species, could also be used to support conservation efforts by permitting the identification of hotspots of donkey biodiversity worldwide, the marker-assisted setup of breeding plans to counter the within-breed loss of variation by genetic drift and to monitor the increase of inbreeding levels.

## Conclusions

When seen as a whole, our findings suggest that, like other terrestrial livestock species, the processes of donkey domestication and subsequent worldwide diffusion were complex, extended over a long period of time, and were influenced by natural, anthropogenic, and environmental factors. In fact, it is difficult to fit all available molecular evidence into a single, simple explanation and additional data, especially from ancient DNA of pre- and early post-domestication specimens from Northeastern Africa and surrounding regions, will be necessary to definitively clarify the origin of the two highly divergent haplogroups and the dynamics of their post-domestication spread. While genomic data seem to support a single wave of expansion out of the domestication center followed by diverging dispersion routes towards Asia and Europe [7], mtDNA data currently show patterns of variation suggesting different expansion timeframes and diffusion trajectories for the two lineages. A number of factors, including founder effects (e.g., the number and frequency of variants belonging to either haplogroup in the founding stocks of different regions) combined with region-specific demographic histories, geographical barriers and migrations along diverse terrestrial and maritime routes can account for the uneven geographical distribution and the predominance of either clade in specific regions. Regarding the African continent, evidence from other livestock species [62] already highlighted a strong geographical sub-structuring of diversity at the nuclear genomic level, particularly the presence of distinct gene pools in Eastern, Western and Northern Africa, which corresponded to different within-continent human migrations routes in pre-historical and historical times. Nuclear genomic evidence recently confirmed this pattern also in donkey populations [6, 7] in which the geographical partitioning of variation strongly mirrors the structuring of mtDNA control-region diversity that we observe within Africa. On the other hand, historical crossroads and continental trade routes fit well with the occurrence of diversity-rich regions in various continents that harbor a significant portion of the global donkey diversity. The new haplogroup nomenclature we proposed bridges the gap between the donkey and other livestock and offers a unified framework for the assessment of mitochondrial variation in this species.

## Supporting information

S1 Table Cycling conditions used for the amplification of the 384 base-pair control-region fragment. This file contains the details of the touch-down PCR cycling conditions used to amplify the 384 bp fragment of the donkey mtDNA control-region analysed in the present work.
(XLSX)

S2 Table Database of mtDNA control-region sequences used in the present study. This file contains all the control-region sequences used to assemble the datasets of the present work.
(XLSX)

S3 Table Table of modified weights of mutated positions adopted for the calculation of Median-joining networks. Modified weights of mutated positions adopted for the calculation of Median-joining networks with Network software.
(DOCX)

S4 Table List of complete mtDNA sequences from GenBank and SRA databases used in the present work. This file contains the database of the complete mtDNA sequences used to assemble the datasets of the coding-region analyses.
(XLSX)

S5 Table Diagnostic mutational motifs of donkey mtDNA haplogroups and sub-haplogroups. Diagnostic mutational motifs of donkey mtDNA haplogroups and sub-haplogroups.
(DOCX)

S6 Table Analysis of molecular variance (AMOVA) performed on the worldwide dataset. Tables summarizing the result of AMOVA analyses performed on the worldwide dataset at different hierarchical levels: a) global dataset: between geographical regions; b) global dataset: between countries; c) global dataset: between clades and between geographical areas within clade; d) global dataset: between clades and between countries within clade; e) haplogroup A: between geographical regions; f) haplogroup B: between geographical regions; g) haplogroup A: between countries; h) haplogroup B: between countries.
(XLSX)

S7 Table Population expansion parameters based on mismatch distribution analysis results. Population expansion parameters based on Mismatch Distribution analysis results. Tau = time since expansion expressed in units of mutational time (Rogers, 1995), Fu’s FS = Fu’s FS index, P(FS) = P value for Fu’s FS index, SSD = Sum of Squared Deviations, P(SSD) = P value for SSD, HRI = Harpending’s Raggedness Index, P(HRI) = P value for Harpending’s Raggedness Index.
(XLSX)

S8 Table Population expansion times based on mismatch distribution analysis results. Population expansion times based on Mismatch Distribution analysis results. Tau = time since expansion expressed in units of mutational time (Rogers, 1995), C.I. 95% = 95% Confidence Interval.
(XLSX)

S1 Fig Boxplot distributions of missing positions and heteroplasmies in sequences from both haplogroups when mapped either to “reference B” (NC_001788.1) or “reference A” (MK982180). (A) Number of missing positions compared to “reference B”; (B) Number of heteroplasmies compared to “reference B”; (C) Number of missing positions compared to “reference A”; (D) Number of heteroplasmies compared to “reference A”. Outliers with more than 60 missing positions or more than 60 heteroplasmies were removed to better visualize the boxplot distribution (outliers were all from clade A: 1 each in panels A, C and D; 4 in panel B). The boxes contain the median (the thick line inside the box), while the lower and upper hinges represent the 25th and 75th percentiles, respectively. The lower and upper whiskers represent the smallest observation greater than or equal to lower hinge minus the interquartile range multiplied by 1.5 and the largest observation less than or equal to upper hinge plus the interquartile range multiplied by 1.5, respectively.
(TIF)

S2 Fig Boxplot distributions of missing positions and heteroplasmies in all sequences from both haplogroups when mapped to their own internal reference sequence. (A) Number of missing positions; (B) Number of heteroplasmies. The boxes contain the median (50%, the thick line inside the box), while the lower and upper hinges represent the 25th and 75th percentiles, respectively. The lower and upper whiskers represent the smallest observation greater than or equal to the lower hinge minus the interquartile range multiplied by 1.5 and the largest observation less than or equal to the upper hinge plus the interquartile range multiplied by 1.5.
(TIF)

S3 Fig Boxplot distributions of missing positions and heteroplasmies in all sequences from both haplogroups when mapped to either reference sequence. (A) Number of missing positions; (B) Number of heteroplasmies. The intercepts represent the upper whisker values of the two distributions for clade A (red dashed line) and for clade B (blue dashed line). The boxes contain the median (50%, the thick line inside the box), while the lower and upper hinges represent the 25th and 75th percentiles, respectively. The lower and upper whiskers represent the smallest observation greater than or equal to the lower hinge minus the interquartile range multiplied by 1.5 and the largest observation less than or equal to the upper hinge plus the interquartile range multiplied by 1.5.
(TIF)

S4 Fig Maximum parsimony tree of 164 coding-region sequences extracted from donkey mitogenomes. The tree encompasses 161 published sequences from domestic donkeys, and one published sequence from Nubian wild ass (*Equus africanus africanus*, KX669267.1), all compared with the donkey reference sequence (DRS, NC_001788.1), and it was rooted by using two published sequences from wild animals (*Equus africanus somaliensis*; MG885769 and KM881681). The control region (D-loop: np 15464–16670) was not considered. Mutations are shown on the branches and are numbered according to DRS. Mutations are transitions unless a base is explicitly indicated. Suffixes indicate transversions (to A, G, C, or T) or reversions (@). Heteroplasmy (het) is indicated. Recurrent mutations are underlined. Insertions and deletions were disregarded. The geographic origin of each sample is indicated with specific colours; wild samples are labelled in the sample name.
(XLSX)

S5 Fig Posterior distributions of the time to the most recent common ancestor and baysian skyline plot of female effective population size. Panel A) posterior distributions of the time to the most recent common ancestors of haplogroups A and B. Labels indicate their means. Panel B) Bayesian skyline plot (BSP) displaying changes in the female effective population size through time considering a generation time of 8 years. The Y axis is in log10 scale.
(TIF)

S6 Fig Median-joining network backbone with control-region haplotype labelling. The picture shows the backbone of the median-joining network depicted in Fig 2 with the alphanumeric codes of control-region haplotypes corresponding to the nodes.
(PDF)

S7 Fig Mismatch distribution plots. Mismatch distributions for the overall dataset (left column) and for sequences of Haplogroup A (central column) and Haplogroup B (right column). The top row refers to the worldwide dataset. The remaining rows refer to the different geographical regions (for label explanations see Fig 2 caption).
(PDF)

S8 Fig Worldwide (a) and regional [b) AFE: Eastern Africa; c) AFN: Northern Africa; d) AFS: Southern Africa; e) AFW: Western Africa; f) AME: America; g) ASA: Arabian Peninsula; h) ASC: Central-Western Asia; i) ASE: Eastern Asia; j) ASS: Southern Asia; k) EUB: the Balkans; l) EUE: Eastern Europe; m) EUI: Italy; n) EUW: Western Europe] median-joining networks of control-region sequences. Regional median-joining networks of control-region sequences obtained with modified mutated positions weights and plotted on the backbone of the global network shown in Fig 2.
(PDF)

S1 Text CPNetwork, a custom script to plot Network software results in R. Custom plot of Network software results in R.
(DOCX)
