# Supplementary material for: Mitochondrial DNA control-region and coding-region data highlight geographically structured diversity and post-domestication population dynamics in worldwide donkeys
Source: PLoS One. 2024 Aug 28;19(8):e0307511. doi: 10.1371/journal.pone.0307511 (PMC11356394; doi:10.1371/journal.pone.0307511)
Supplement: S3 Table — Modified weights of mutated positions adopted for the calculation of Median-joining networks with Network software. (DOCX) [file pone.0307511.s003.docx]

**Additional file 3 Table S3**

**Weight given to the different control-region mutations, based on mutation type and frequency.**

Genetic relationships between mtDNA haplotypes were reconstructed using Median-Joining Networks (MJN) with Network v.5.0 software (Bandelt et al. 1999). The weight of each mutation in the Network software default settings is 10. Based on the mutation type and frequency, different weights were assigned to some mutations: transitions recurring many times within a single haplogroup, weight = 0; transitions recurring in the overall network, weight = 1 (more frequent), 3, or 5 (less frequent); transversions, weight = 13; insertions, weight = 15; transitions, transversions or insertions at the same position but in different haplogroups, weight = 20.

| **Position** | **Weight** |
| --- | --- |
| 15476 | 5 |
| 15480 | 15 |
| 15489 | 15 |
| 15490 | 20 |
| 15520 | 13 |
| 15522 | 13 |
| 15525 | 13 |
| 15527 | 13 |
| 15529INS | 13 |
| 15531 | 13 |
| 15532 | 13 |
| 15541 | 13 |
| 15560 | 13 |
| 15569 | 5 |
| 15580 | 5 |
| 15598 | 1 |
| 15599 | 5 |
| 15621 | 0 |
| 15628 | 3 |
| 15637 | 15 |
| 15645 | 0 |
| 15662 | 3 |
| 15667 | 3 |
| 15668 | 13 |
| 15677 | 13 |
| 15691 | 13 |
| 15699INS | 13 |
| 15715 | 13 |
| 15727 | 13 |
