# Supplementary material for: Mitochondrial DNA control-region and coding-region data highlight geographically structured diversity and post-domestication population dynamics in worldwide donkeys
Source: PLoS One. 2024 Aug 28;19(8):e0307511. doi: 10.1371/journal.pone.0307511 (PMC11356394; doi:10.1371/journal.pone.0307511)
Supplement: S5 Table — Diagnostic mutational motifs of donkey mtDNA haplogroups and sub-haplogroups. (DOCX) [file pone.0307511.s005.docx]

**Additional file 10 Table S5.** Diagnostic mutational motifs of donkey mtDNA haplogroups and sub-haplogroups.

| **Clade** | **Diagnostic Coding-Region Mutational Motif^a, b, c^** |
| --- | --- |
| **A** | 466, 706, 956, 1425, 2376, 3107, 3128, 3398, 3587, 3648, 3859, 3961, 4081, 4243, 4744, 4801, 4815, 4861, 4954, 5494, 5497T, 5566, 5824, 6034, 6097, 6100, 6505, 7162, 7573, 7920, 8194, 8197, 8377, 8638, 8817, 9027, 9144A, 9686, 9716, 9821, 10492, 10777, 10798, 11011, 11113, 11203, 11461, 11747, 11771, 12301, 12356, 12442, 12469, 12805, 13108, 13159, 13306G, 13401, 13620, 13815, 13829, 14289, 14409, 14421, 14673, 14883, 15187, 15318 |
| **AB** | 223, 432, 489, 824, 977, 2540, 3152, 3407, 4387, 4505, 4699, 5014, 5248, 5365, 6019, 6688, 7028, 7312, 7426, 7743T, 8053, 8332, 8350, 8629, 8767, 8874, 9010, 10243, 11056, 11131, 11182, 11809, 12424, 12721, 12760, 12794, 12874, 12910, 13015, 13363, 14022, 15099, 15198 |
| **B** | 981, 4759, 5761, 7639, 8135, 9791, 10948 |
| **B1** | 222C, 3428, 8137, 10122G, 10123C, 10839, 12214, 12445, 12855, 13742C |
| **B2^d^** | 1885, 2669A, 2740, 3236, 3404, 3814, 4684, 4744, 5360, 6481T, 7979, 8125, 8426, 10112, 10612, 11203, 11984, 12694, 13691, 14043, 15156 |

^a^ Coding-region mutational motifs are relative to the reference sequence (DRS, GenBank NC_001788), which is a member of haplogroup B.

^b^ Recurrent mutations are underlined.

^c^ The Ancestral Mitogenome of domestic donkeys corresponds to AB.

^d^ An accurate diagnostic motif cannot be determined yet due to the availability of only one sequence.
