# Supplementary figures and images for: Mitochondrial DNA control-region and coding-region data highlight geographically structured diversity and post-domestication population dynamics in worldwide donkeys
Source: PLoS One. 2024 Aug 28;19(8):e0307511. doi: 10.1371/journal.pone.0307511 (PMC11356394; doi:10.1371/journal.pone.0307511)

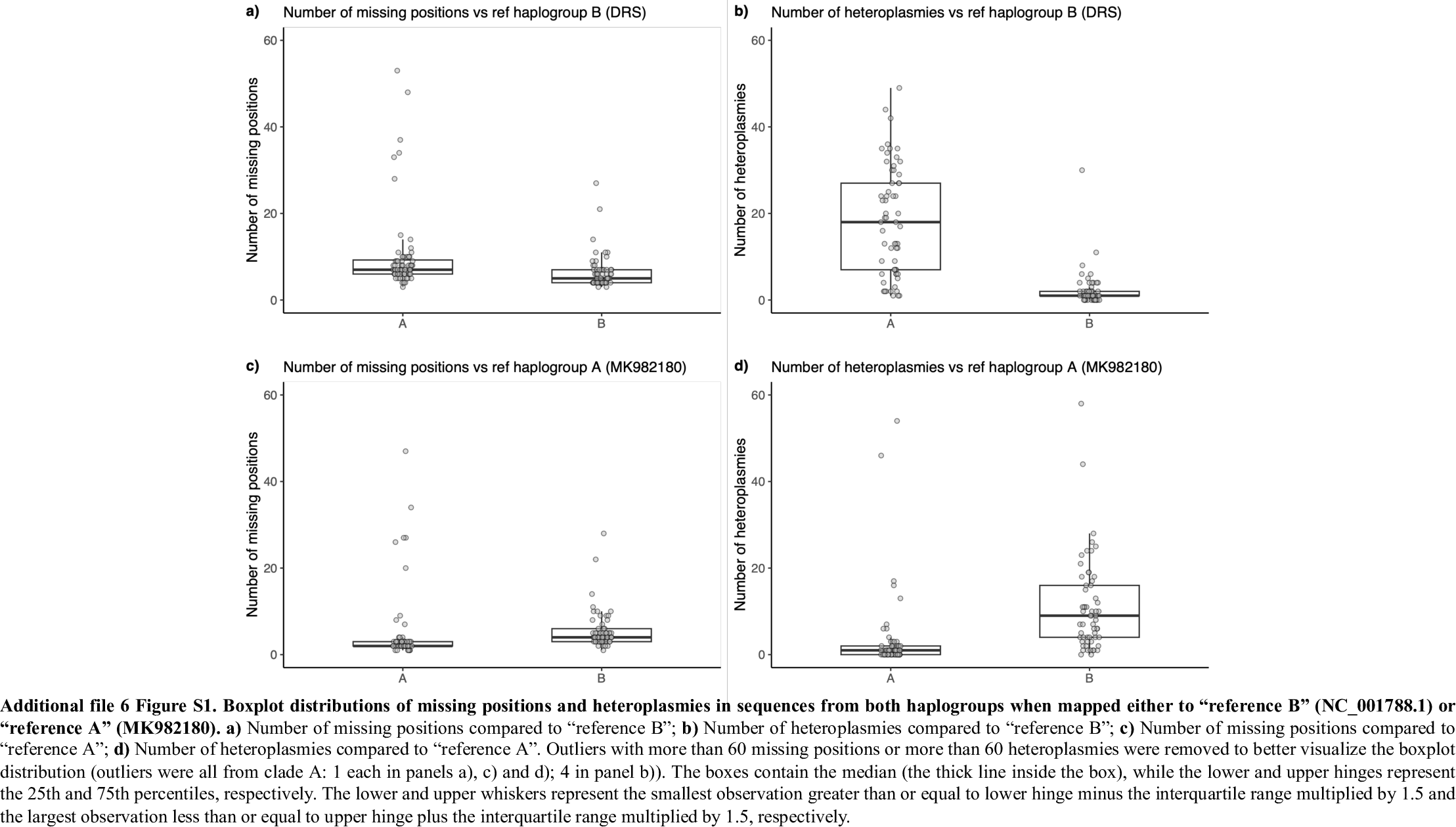

Supplement: S1 Fig — (A) Number of missing positions compared to “reference B”; (B) Number of heteroplasmies compared to “reference B”; (C) Number of missing positions compared to “reference A”; (D) Number of heteroplasmies compared to “reference A”. Outliers with more than 60 missing positions or more than 60 heteroplasmies were removed to better visualize the boxplot distribution (outliers were all from clade A: 1 each in panels A, C and D; 4 in panel B). The boxes contain the median (the thick line inside the box), while the lower and upper hinges represent the 25th and 75th percentiles, respectively. The lower and upper whiskers represent the smallest observation greater than or equal to lower hinge minus the interquartile range multiplied by 1.5 and the largest observation less than or equal to upper hinge plus the interquartile range multiplied by 1.5, respectively. (TIF) [file pone.0307511.s009.tif]

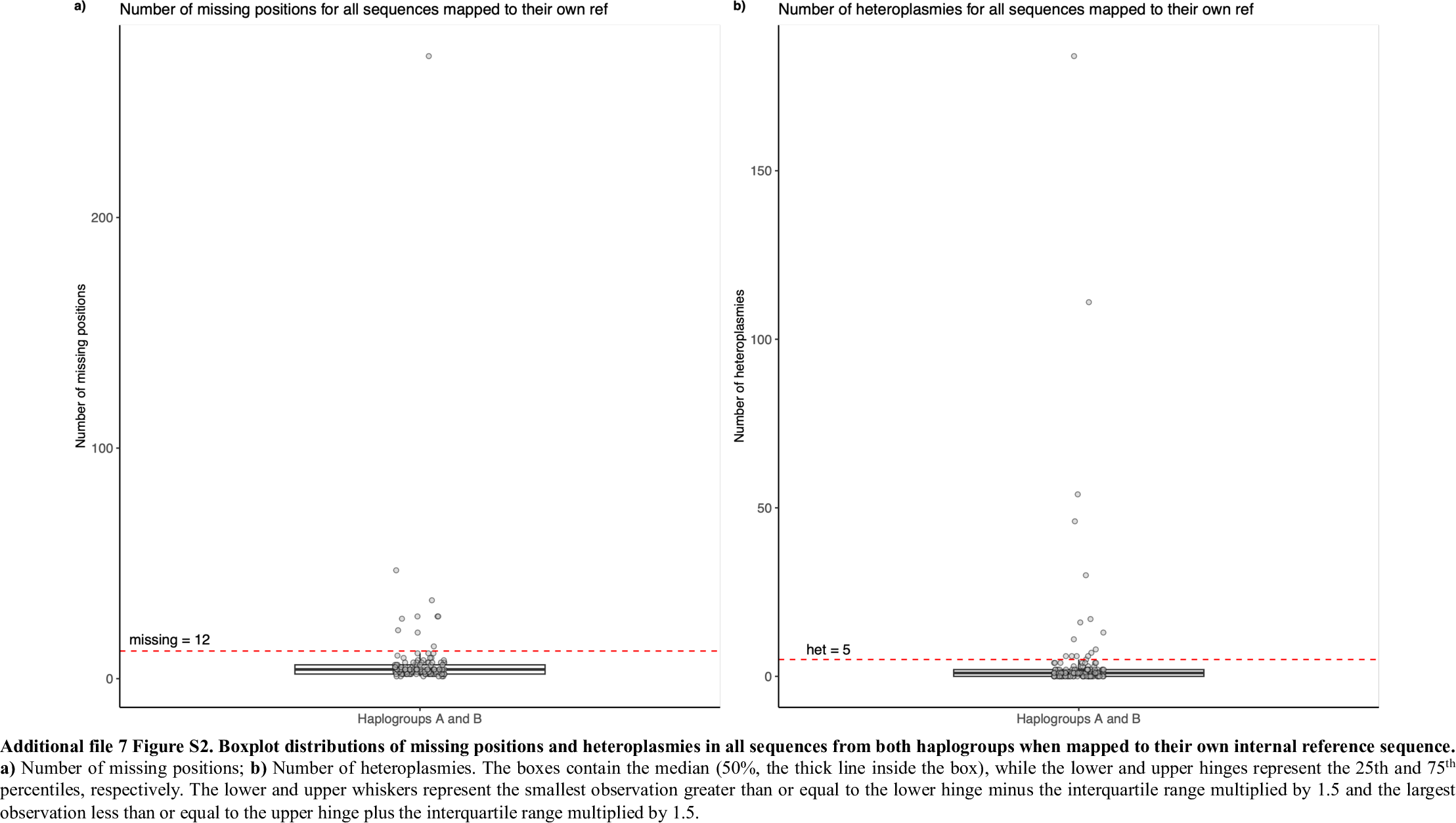

Supplement: S2 Fig — (A) Number of missing positions; (B) Number of heteroplasmies. The boxes contain the median (50%, the thick line inside the box), while the lower and upper hinges represent the 25th and 75th percentiles, respectively. The lower and upper whiskers represent the smallest observation greater than or equal to the lower hinge minus the interquartile range multiplied by 1.5 and the largest observation less than or equal to the upper hinge plus the interquartile range multiplied by 1.5. (TIF) [file pone.0307511.s010.tif]

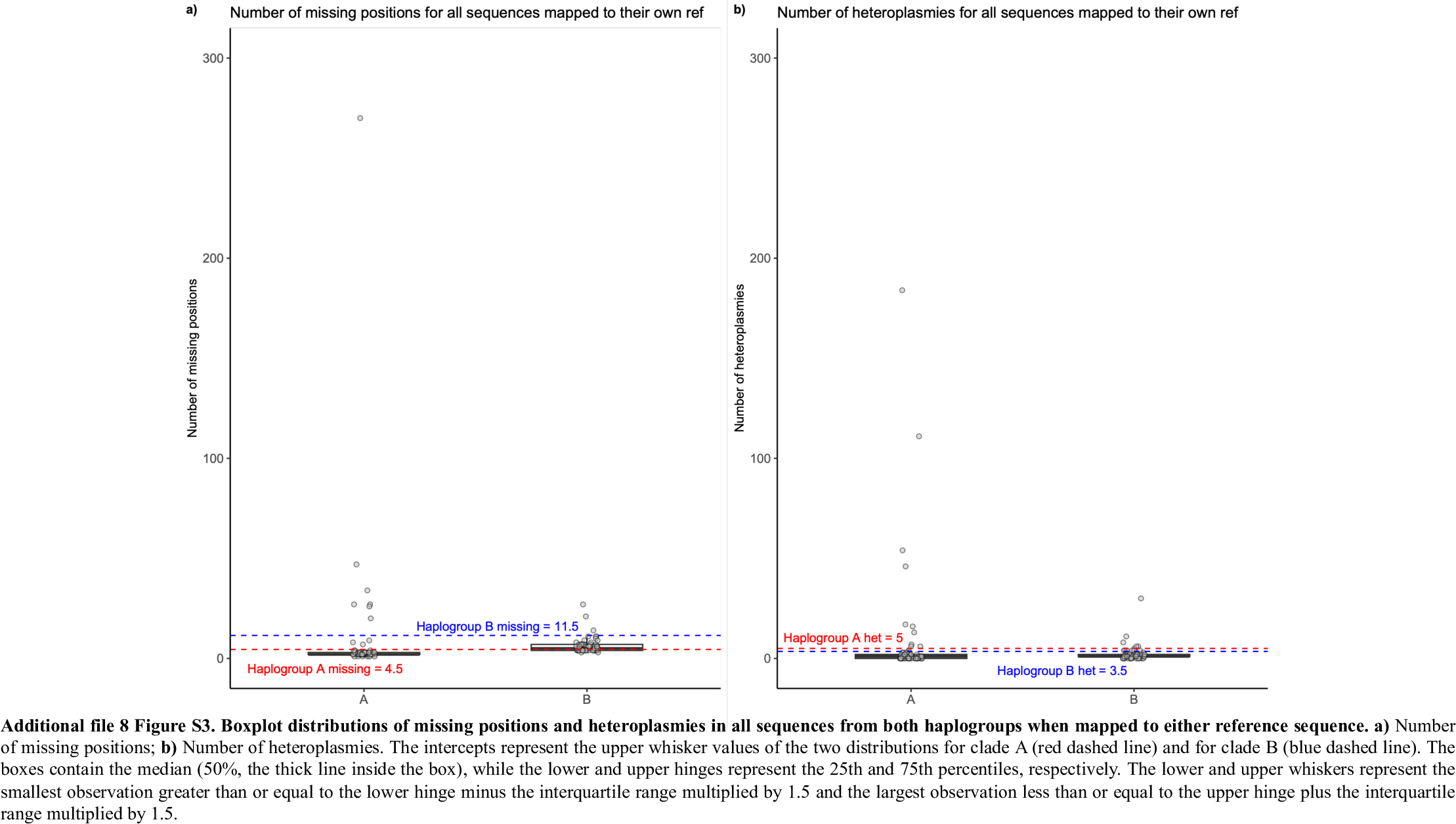

Supplement: S3 Fig — (A) Number of missing positions; (B) Number of heteroplasmies. The intercepts represent the upper whisker values of the two distributions for clade A (red dashed line) and for clade B (blue dashed line). The boxes contain the median (50%, the thick line inside the box), while the lower and upper hinges represent the 25th and 75th percentiles, respectively. The lower and upper whiskers represent the smallest observation greater than or equal to the lower hinge minus the interquartile range multiplied by 1.5 and the largest observation less than or equal to the upper hinge plus the interquartile range multiplied by 1.5. (TIF) [file pone.0307511.s011.tif]

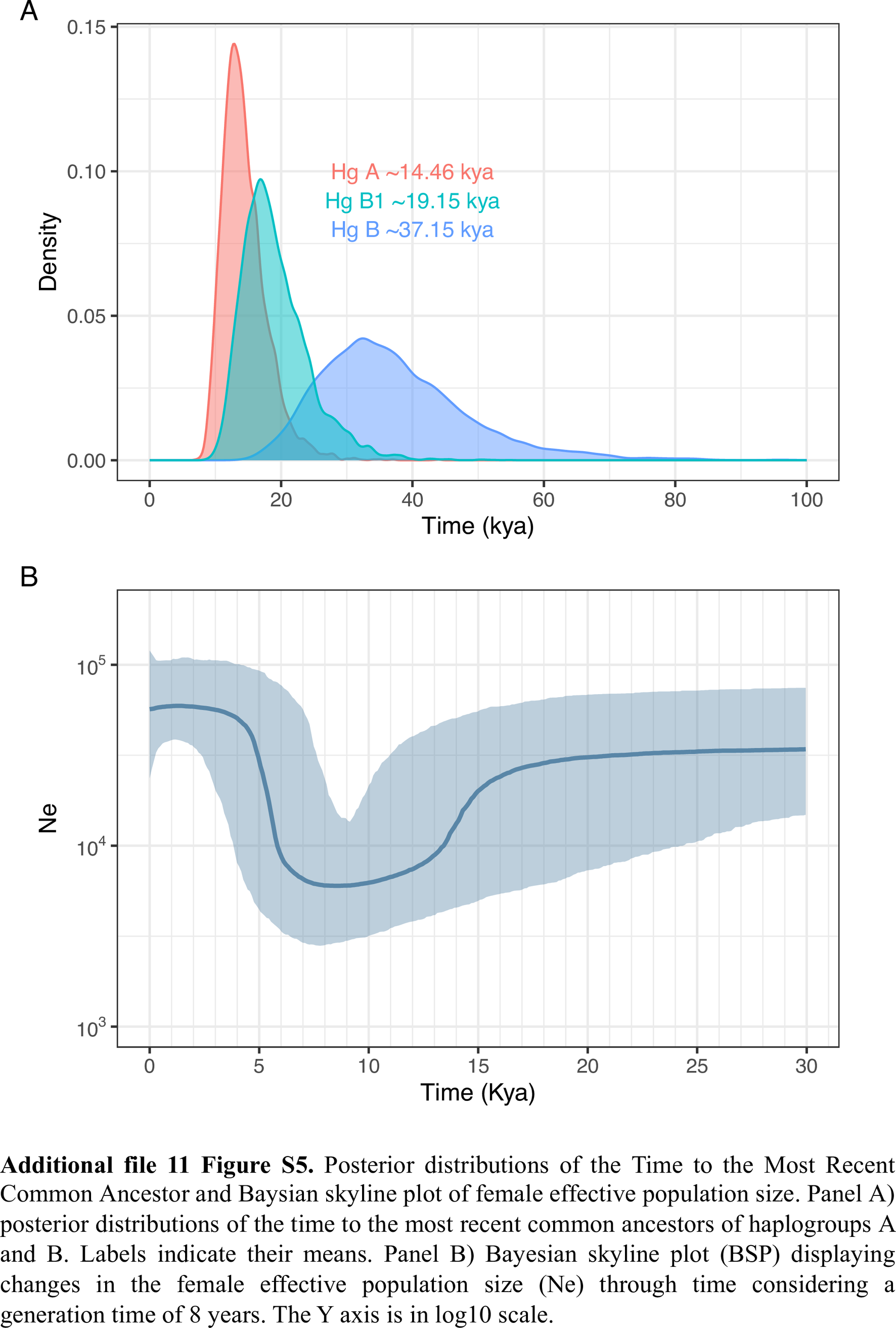

Supplement: S5 Fig — Panel A) posterior distributions of the time to the most recent common ancestors of haplogroups A and B. Labels indicate their means. Panel B) Bayesian skyline plot (BSP) displaying changes in the female effective population size through time considering a generation time of 8 years. The Y axis is in log10 scale. (TIF) [file pone.0307511.s013.tif]
