# Supplementary material for: Mitochondrial DNA control-region and coding-region data highlight geographically structured diversity and post-domestication population dynamics in worldwide donkeys
Source: PLoS One. 2024 Aug 28;19(8):e0307511. doi: 10.1371/journal.pone.0307511 (PMC11356394; doi:10.1371/journal.pone.0307511)
Supplement: S7 Fig — Mismatch distributions for the overall dataset (left column) and for sequences of Haplogroup A (central column) and Haplogroup B (right column). The top row refers to the worldwide dataset. The remaining rows refer to the different geographical regions (for label explanations see Fig 2 caption). (PDF) [file pone.0307511.s015.pdf]

Additional file 14 Figure S7. Mismatch distribution plots. Mismatch distributions for the overall dataset (left column) and for sequences of Haplogroup A (central column) and Haplogroup B (right column). The top row refers to the worldwide dataset. The remaining rows refer to the different geographical regions (for label explanations see Figure 2 caption).

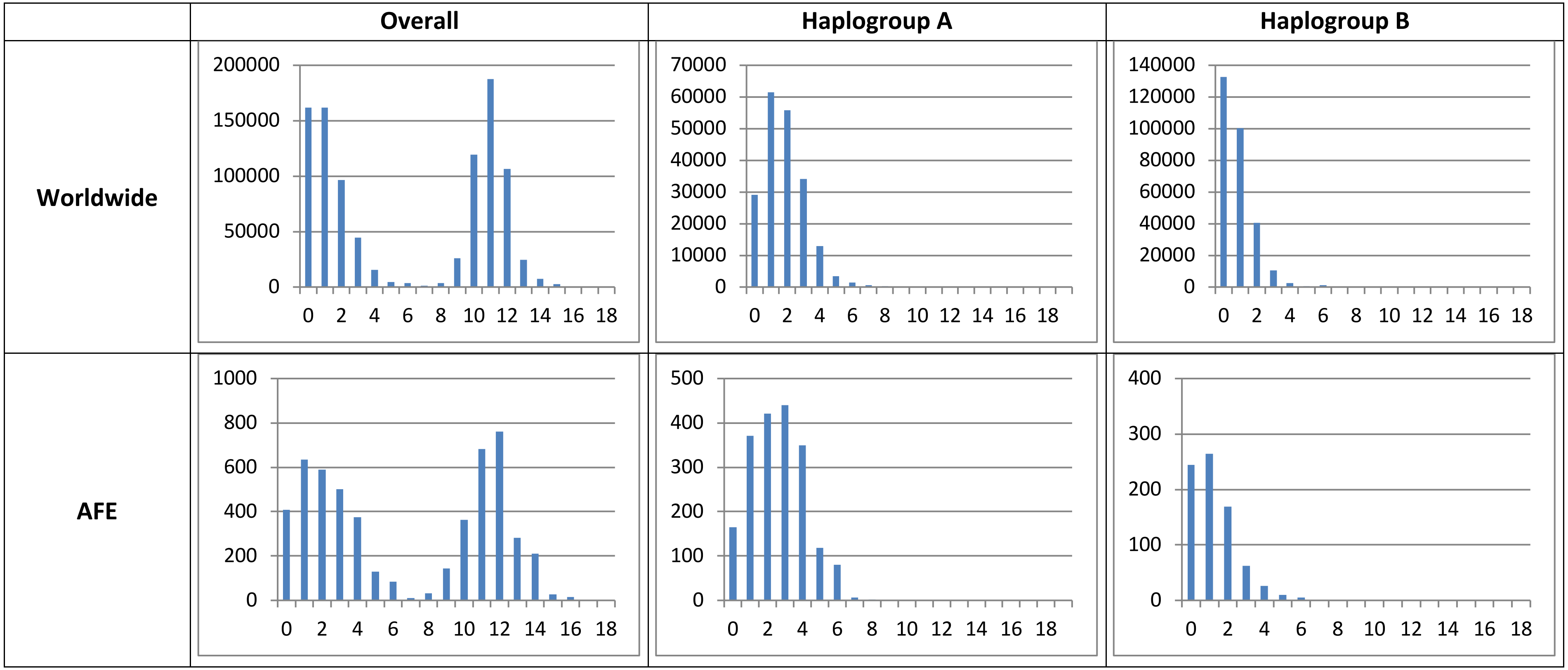

<table>
<tr><td>AFN</td>
<td>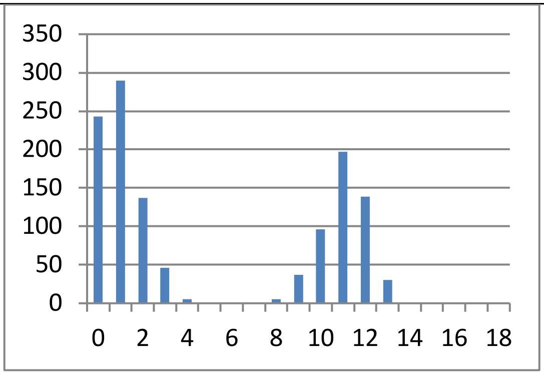
</td>
<td>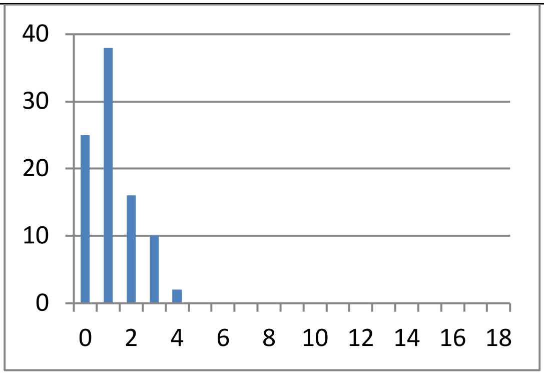
</td>
<td>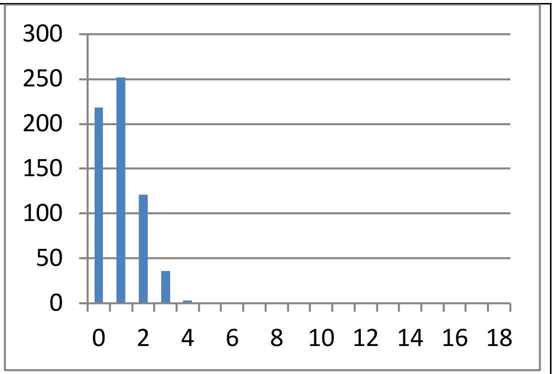
</td></tr>
<tr><td>AFS</td>
<td>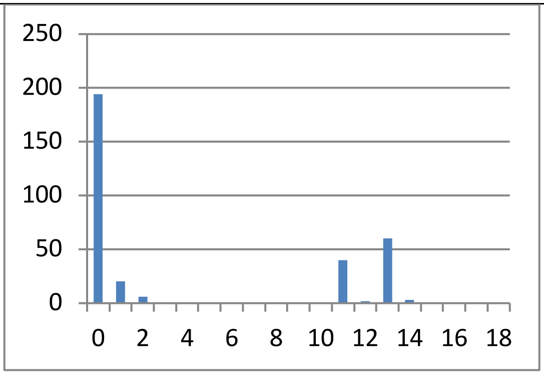
</td>
<td>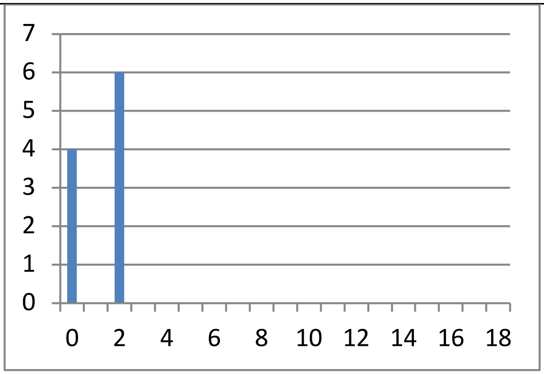
</td>
<td>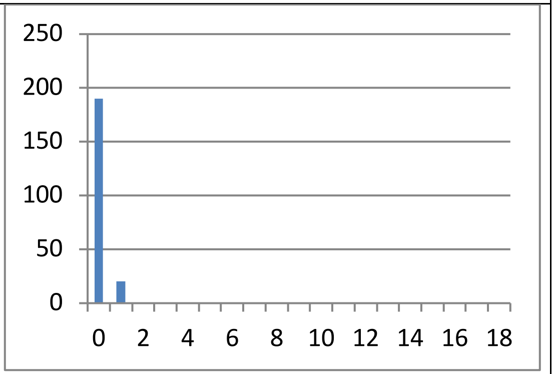
</td></tr>
<tr><td>AFW</td>
<td>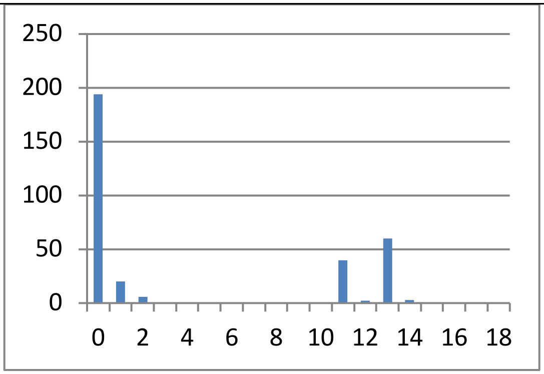
</td>
<td>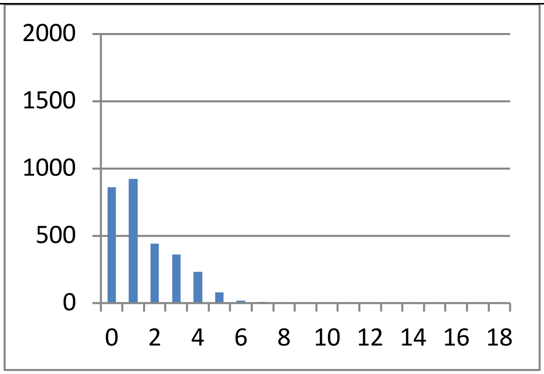
</td>
<td>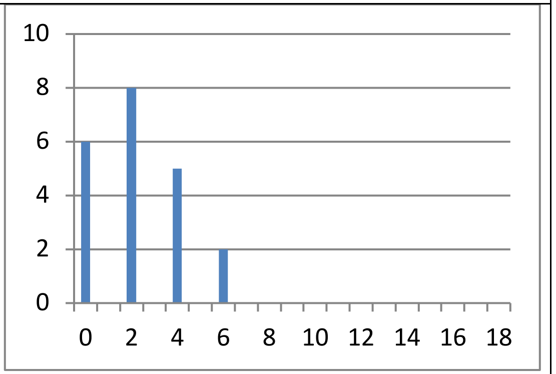
</td></tr>
</table>

**AME**	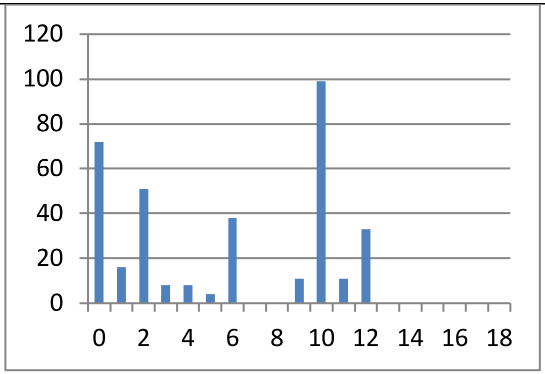0 20 40 60 80 100 120<br>0 2 4 6 8 10 12 14 16 18	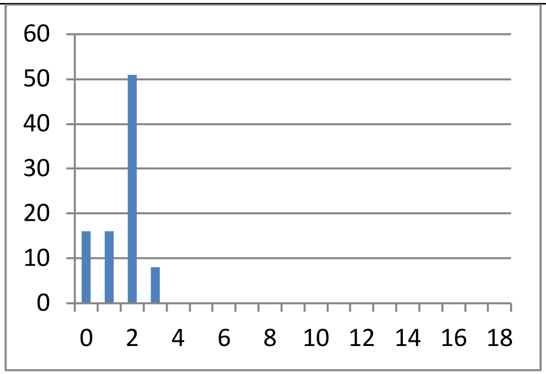0 10 20 30 40 50 60<br>0 2 4 6 8 10 12 14 16 18	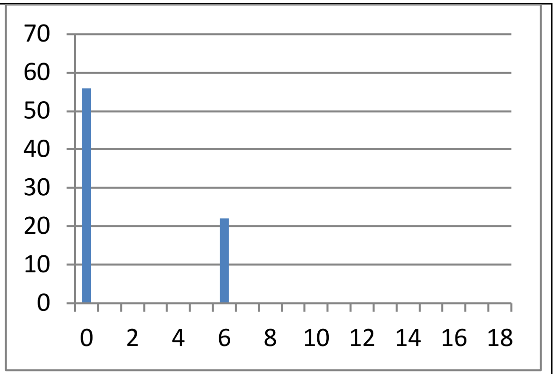0 10 20 30 40 50 60 70<br>0 2 4 6 8 10 12 14 16 18
**ASA**	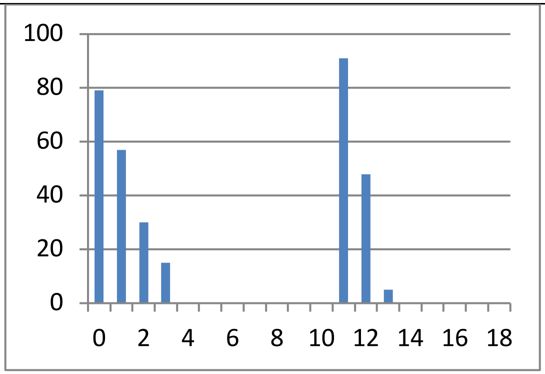0 20 40 60 80 100<br>0 2 4 6 8 10 12 14 16 18	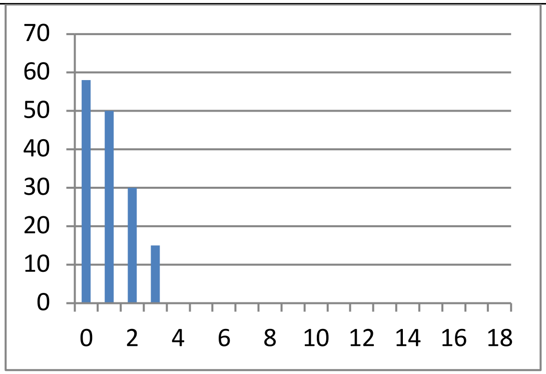0 10 20 30 40 50 60 70<br>0 2 4 6 8 10 12 14 16 18	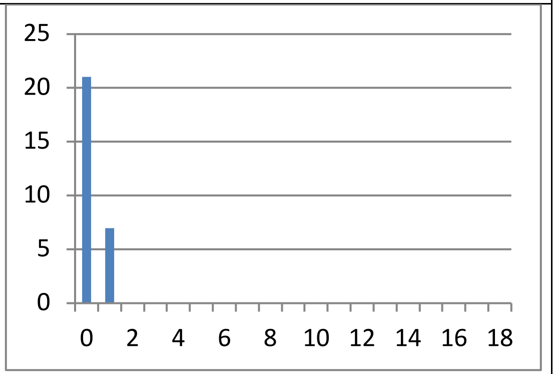0 5 10 15 20 25<br>0 2 4 6 8 10 12 14 16 18
**ASC**	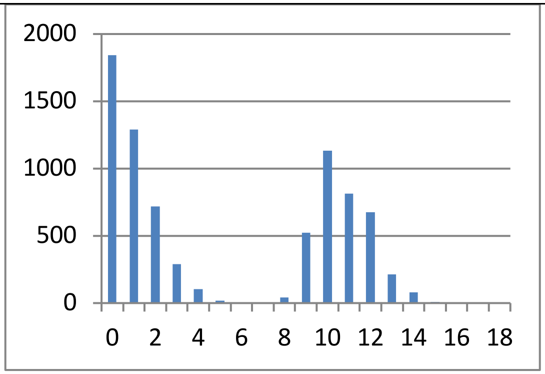0 500 1000 1500 2000<br>0 2 4 6 8 10 12 14 16 18	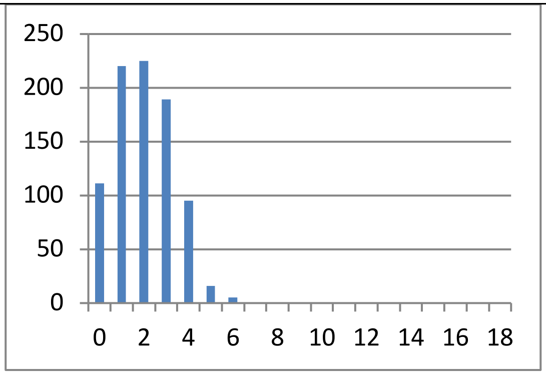0 50 100 150 200 250<br>0 2 4 6 8 10 12 14 16 18	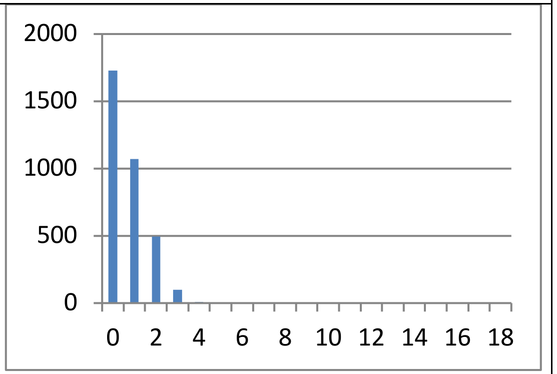0 500 1000 1500 2000<br>0 2 4 6 8 10 12 14 16 18

**ASE**	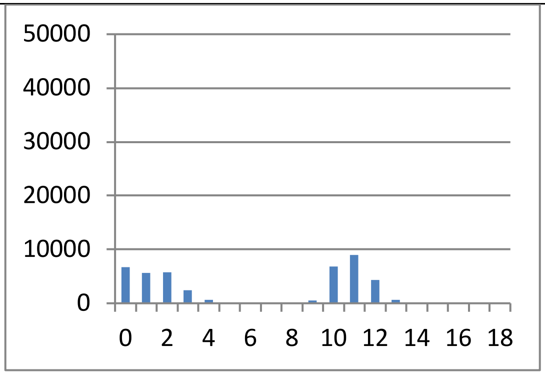	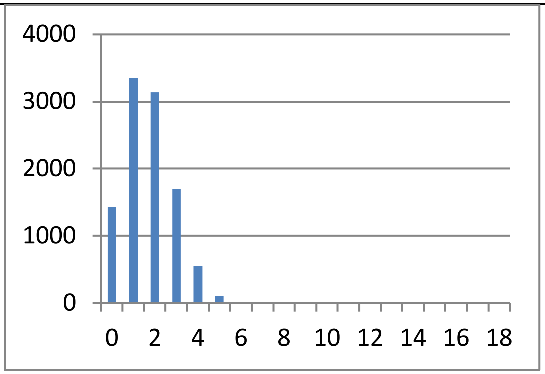	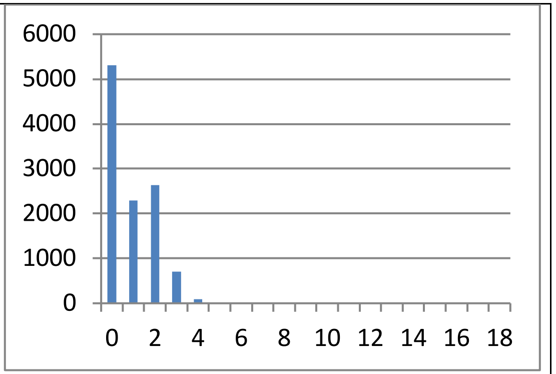
**ASS**	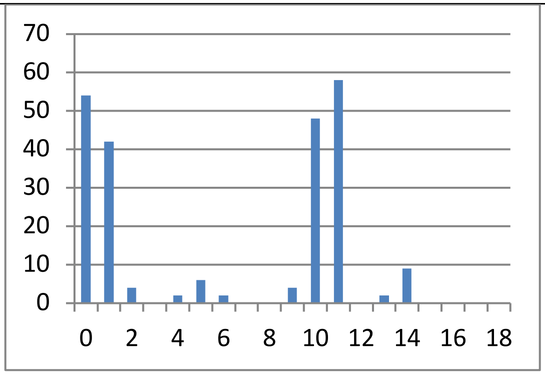	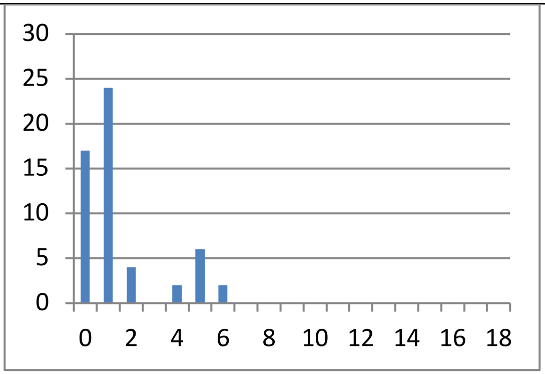	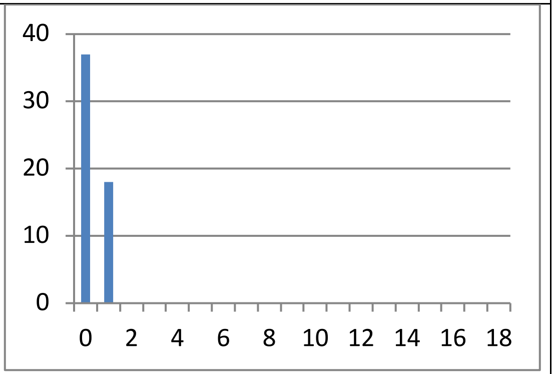
**EUB**	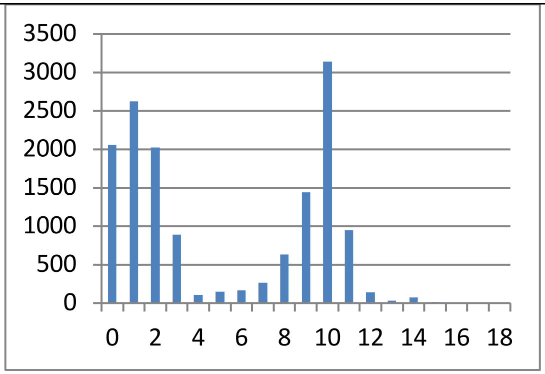	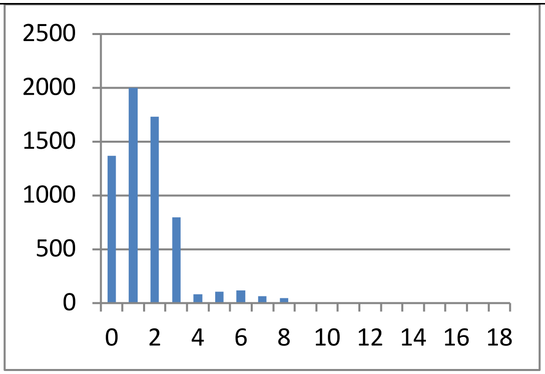	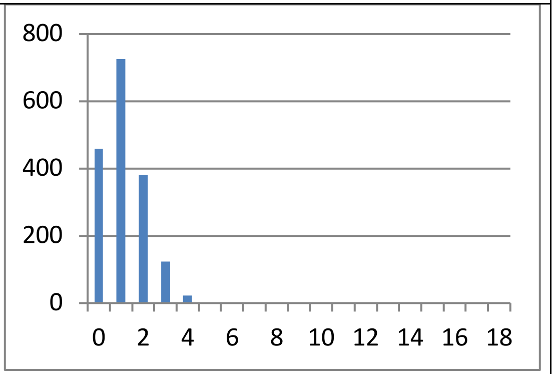

EUE

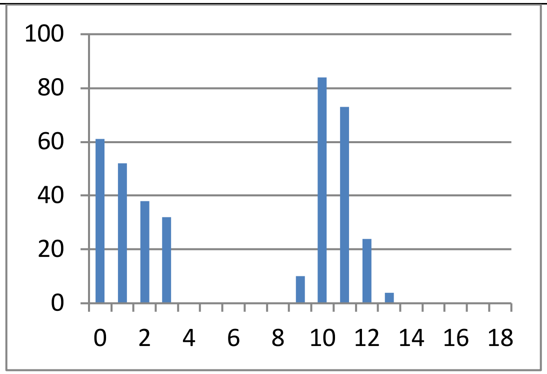
100 80 60 40 20 0
0 2 4 6 8 10 12 14 16 18

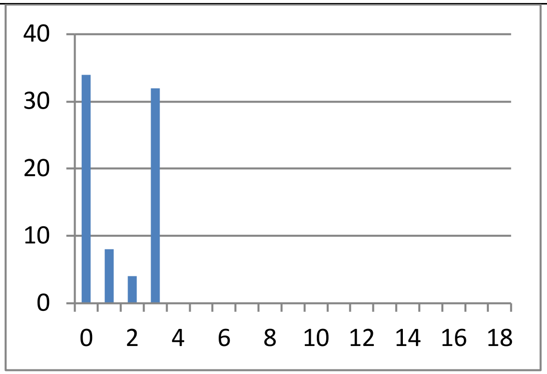
40 30 20 10 0
0 2 4 6 8 10 12 14 16 18

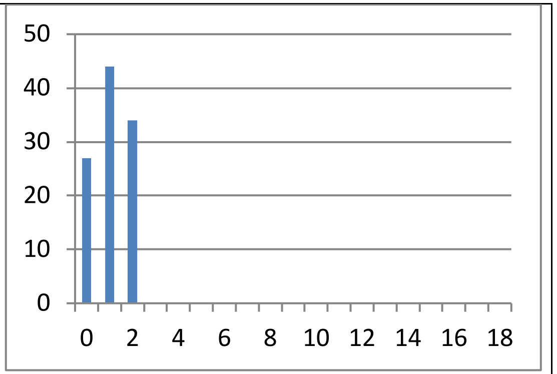
50 40 30 20 10 0
0 2 4 6 8 10 12 14 16 18

EUI

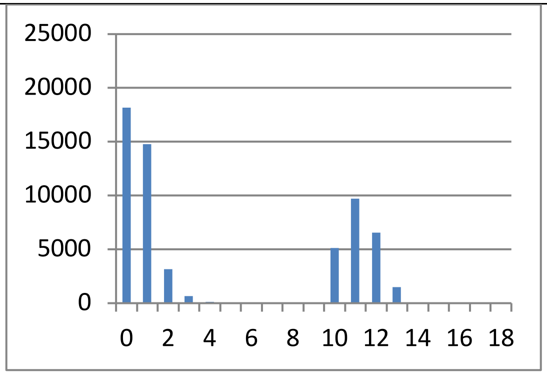
25000 20000 15000 10000 5000 0
0 2 4 6 8 10 12 14 16 18

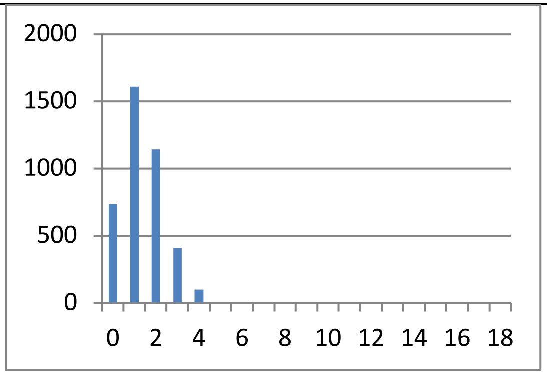
2000 1500 1000 500 0
0 2 4 6 8 10 12 14 16 18

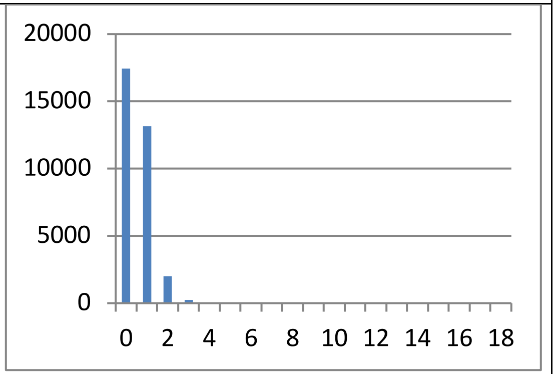
20000 15000 10000 5000 0
0 2 4 6 8 10 12 14 16 18

EUW

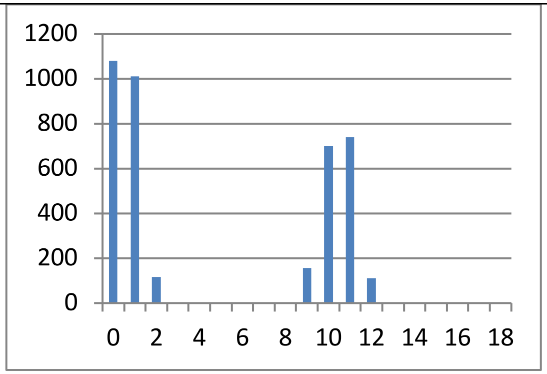
1200 1000 800 600 400 200 0
0 2 4 6 8 10 12 14 16 18

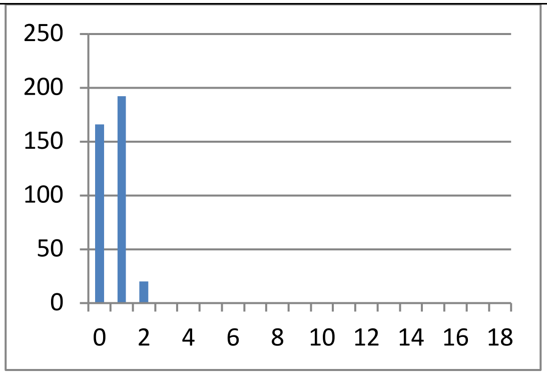
250 200 150 100 50 0
0 2 4 6 8 10 12 14 16 18

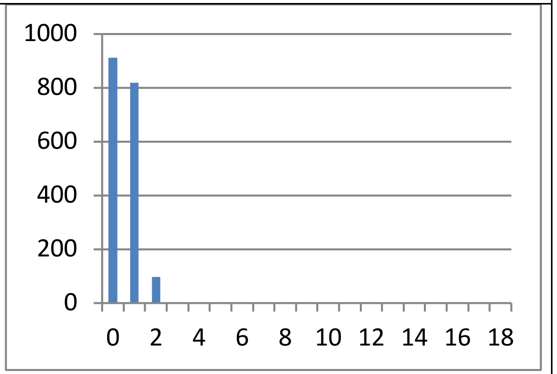
1000 800 600 400 200 0
0 2 4 6 8 10 12 14 16 18
