# Supplementary material for: Mitochondrial DNA control-region and coding-region data highlight geographically structured diversity and post-domestication population dynamics in worldwide donkeys
Source: PLoS One. 2024 Aug 28;19(8):e0307511. doi: 10.1371/journal.pone.0307511 (PMC11356394; doi:10.1371/journal.pone.0307511)
Supplement: S8 Fig — Worldwide (a) and regional [b) AFE: Eastern Africa; c) AFN: Northern Africa; d) AFS: Southern Africa; e) AFW: Western Africa; f) AME: America; g) ASA: Arabian Peninsula; h) ASC: Central-Western Asia; i) ASE: Eastern Asia; j) ASS: Southern Asia; k) EUB: the Balkans; l) EUE: Eastern Europe; m) EUI: Italy; n) EUW: Western Europe] median-joining networks of control-region sequences. Regional median-joining networks of control-region sequences obtained with modified mutated positions weights and plotted on the backbone of the global network shown in Fig 2. (PDF) [file pone.0307511.s016.pdf]

a)

Worldwide network (n=1392)

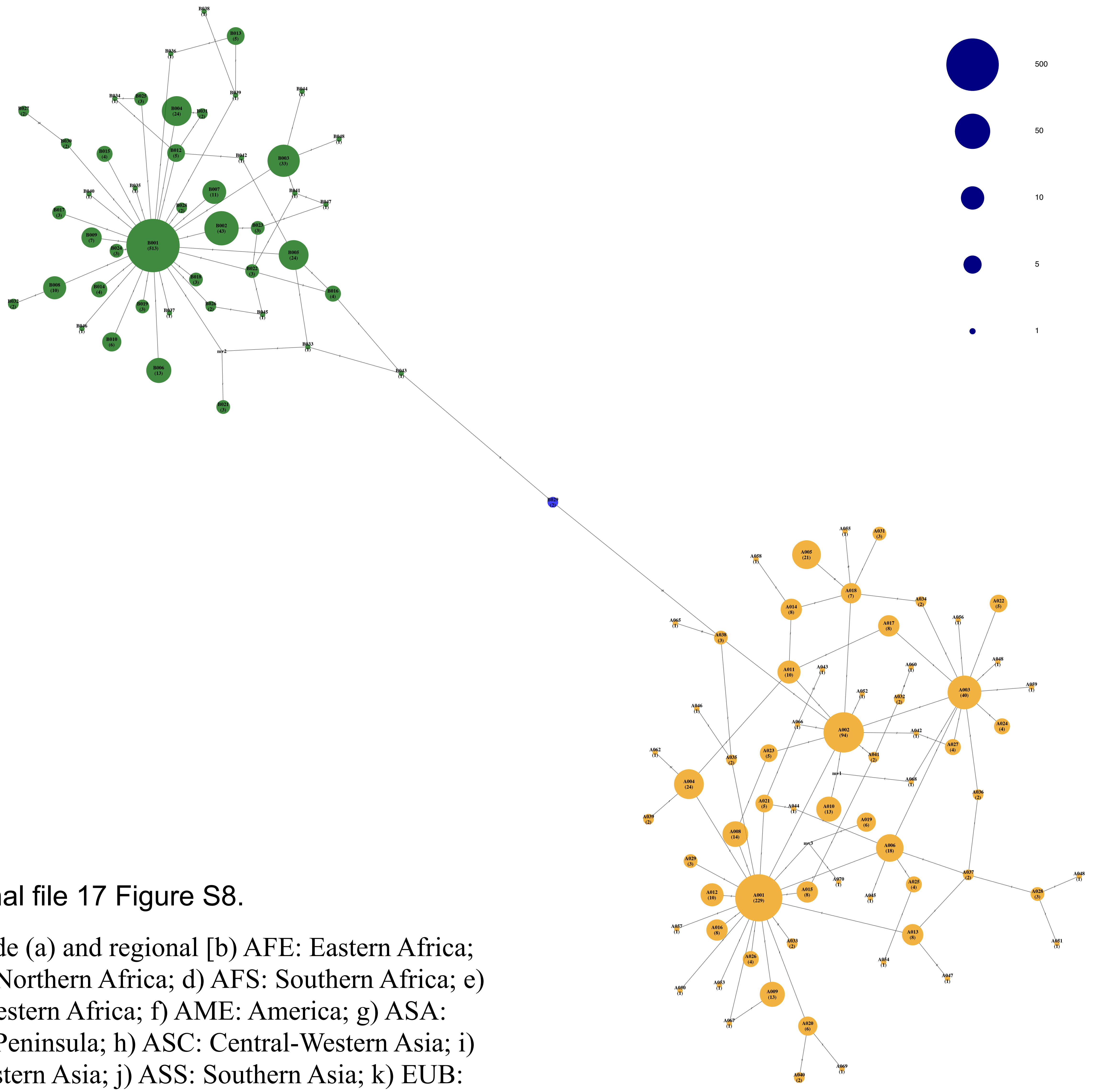

Additional file 17 Figure S8.

Worldwide (a) and regional [b) AFE: Eastern Africa; c) AFN: Northern Africa; d) AFS: Southern Africa; e) AFW: Western Africa; f) AME: America; g) ASA: Arabian Peninsula; h) ASC: Central-Western Asia; i) ASE: Eastern Asia; j) ASS: Southern Asia; k) EUB: the Balkans; l) EUE: Eastern Europe; m) EUI: Italy; n) EUW: Western Europe] median-joining networks of control-region sequences.

b)

AFE (n=103)

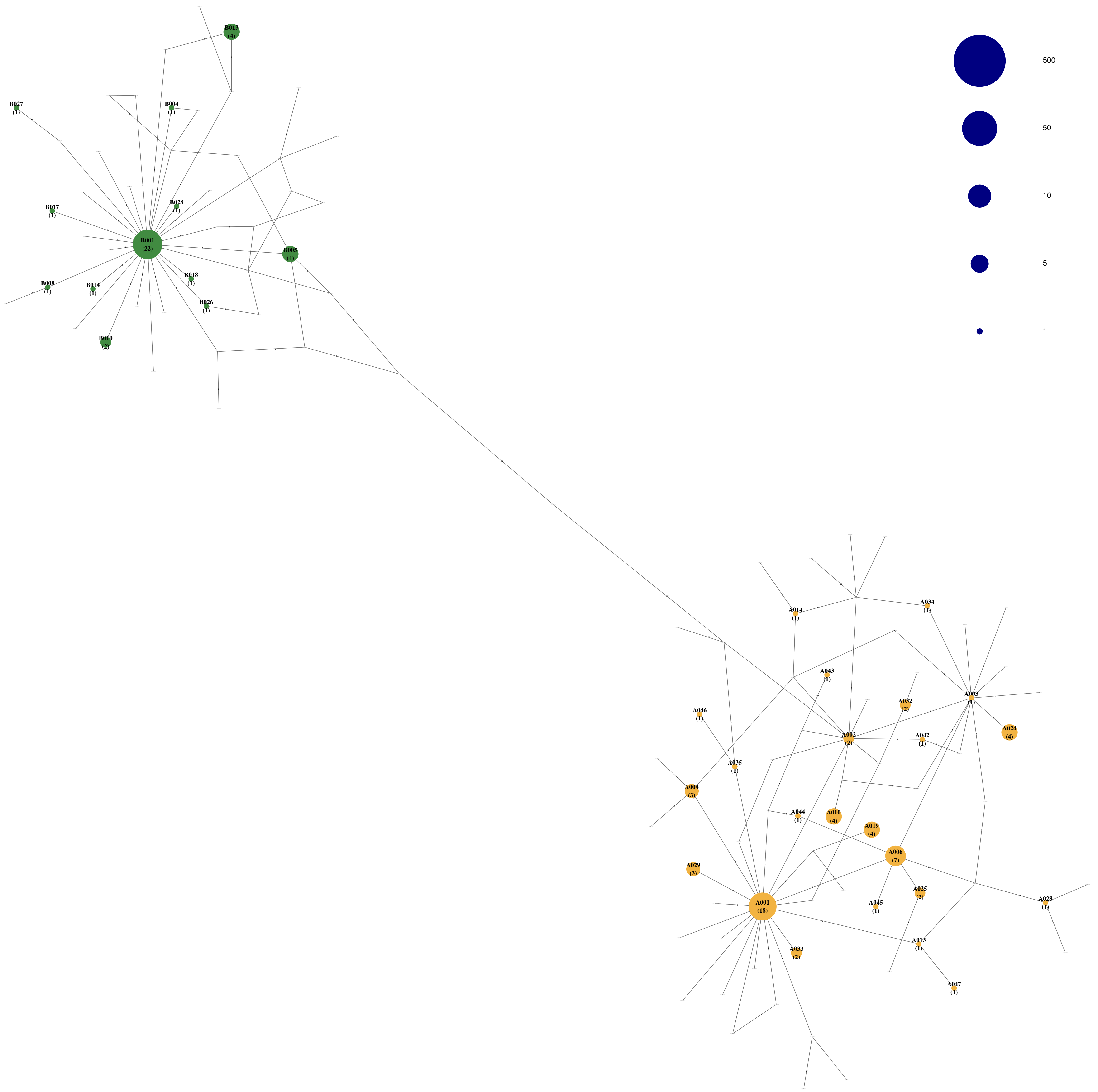

B013 (4)
B027 (1)
B004 (1)
B017 (1)
B028 (1)
B001 (22)
B005 (4)
B008 (1)
B014 (1)
B018 (1)
B026 (1)
B010 (2)
500
50
10
5
1
A014 (1)
A034 (1)
A043 (1)
A003 (1)
A032 (2)
A046 (1)
A002 (2)
A042 (1)
A024 (4)
A035 (1)
A004 (3)
A044 (1)
A010 (4)
A019 (4)
A006 (7)
A029 (3)
A001 (18)
A025 (2)
A045 (1)
A028 (1)
A013 (1)
A033 (2)
A047 (1)

c)

AFN (n=50)

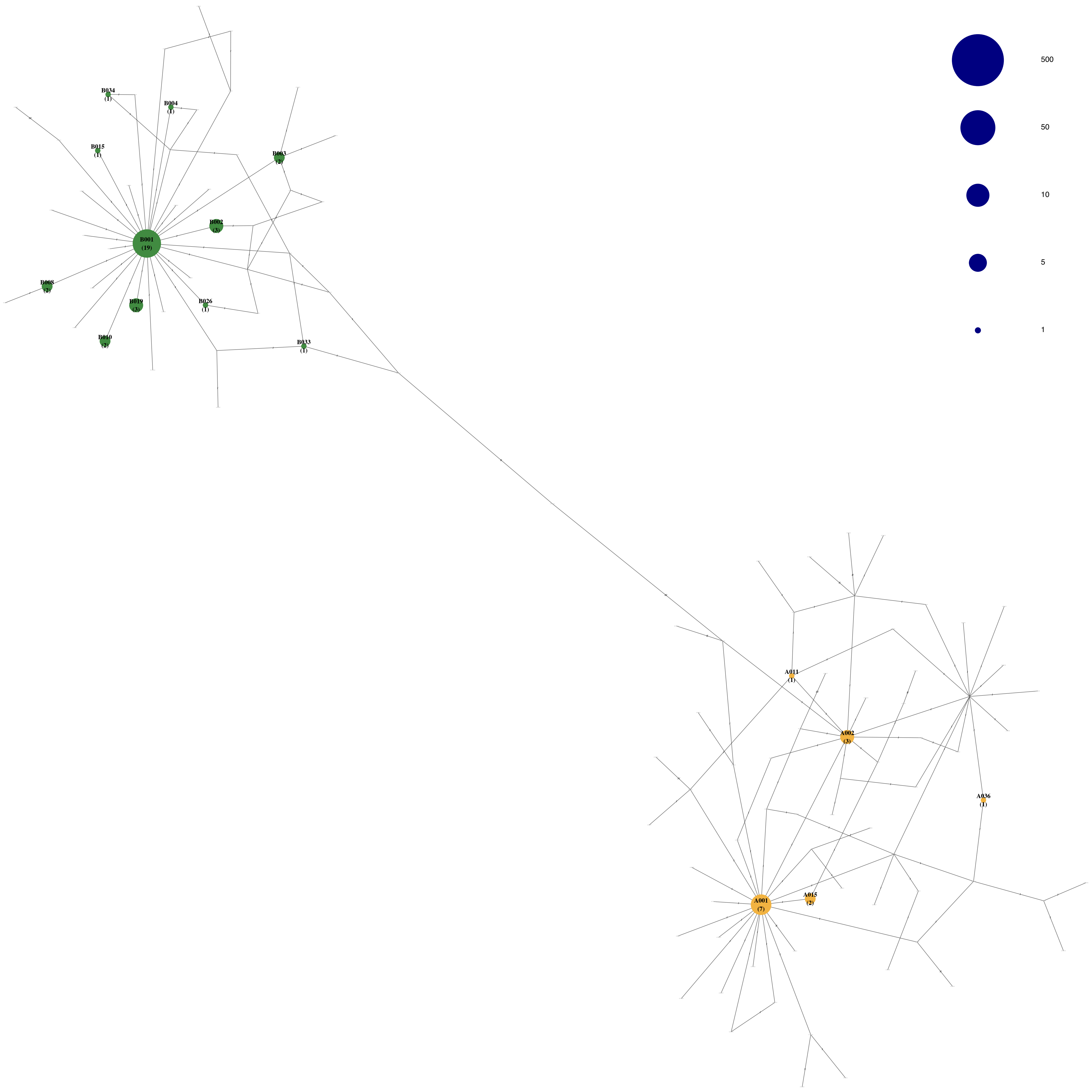

B034
(1)
B004
(1)
B015
(1)
B003
(2)
B002
(3)
B001
(19)
B008
(2)
B019
(3)
B026
(1)
B010
(2)
B033
(1)
A011
(1)
A002
(3)
A036
(1)
A001
(7)
A015
(2)
500
50
10
5
1

d)

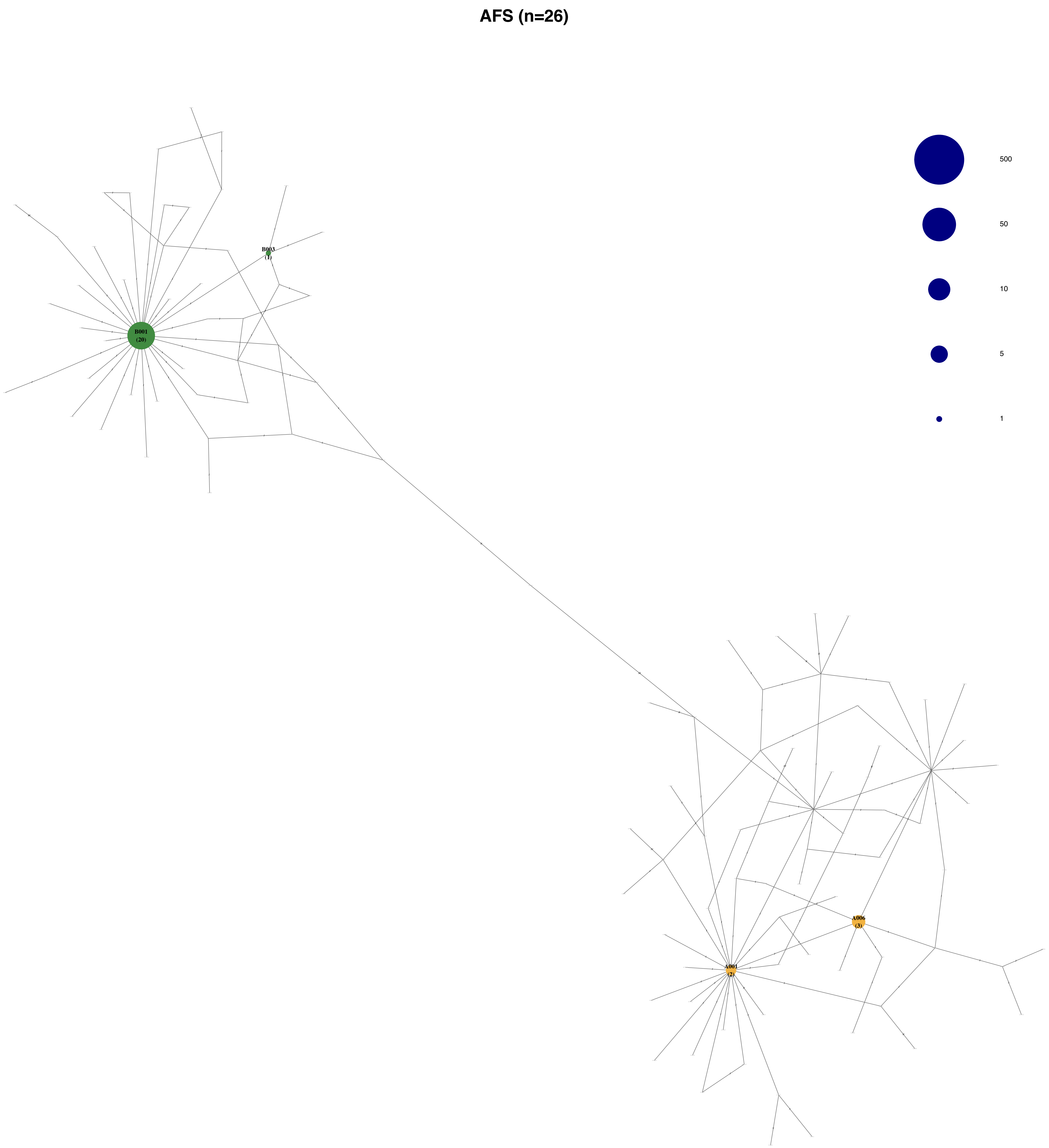

e)

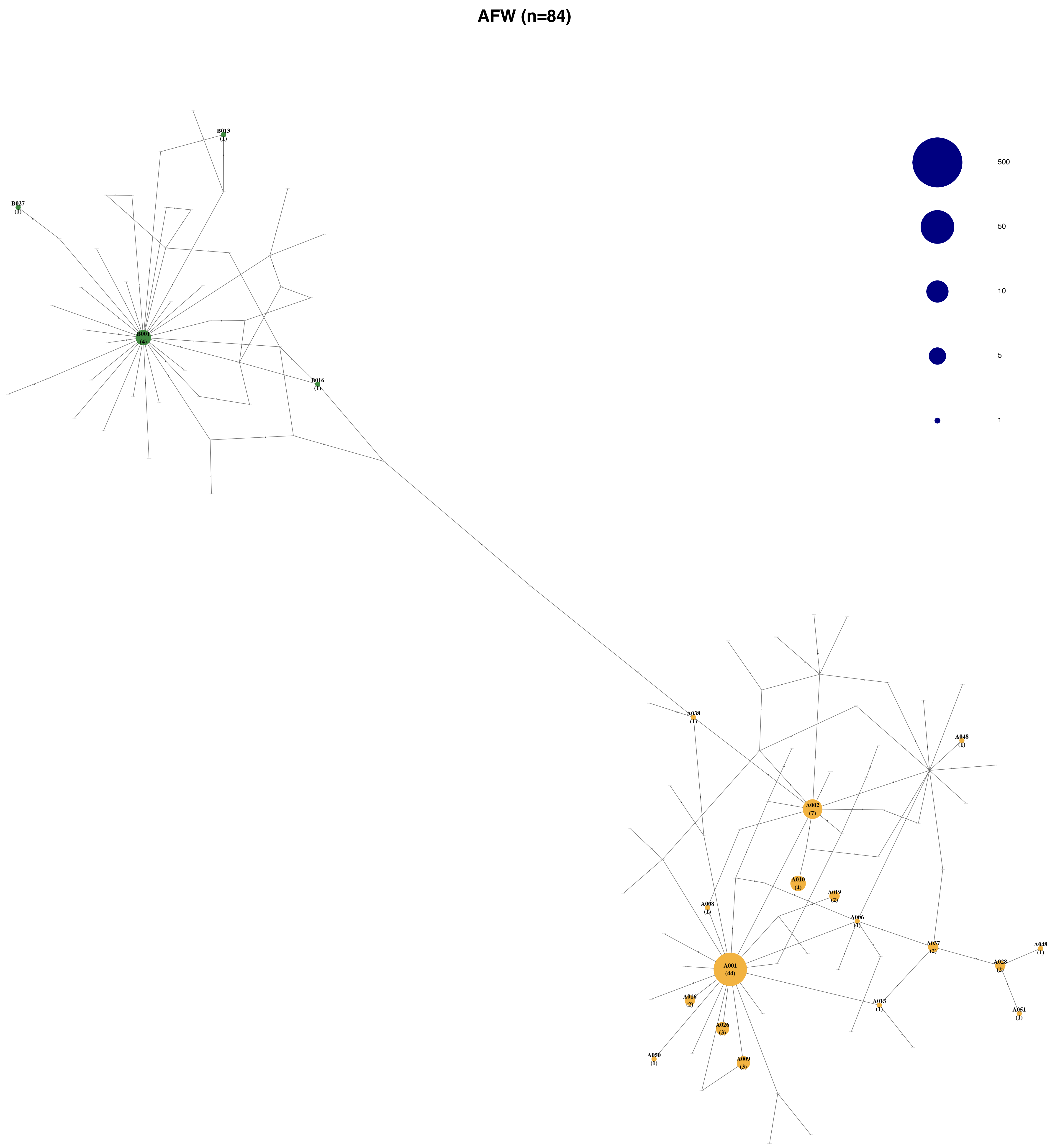

AFW (n=84)
B013
(1)
B027
(1)
B001
(4)
B016
(1)
500
50
10
5
1
A038
(1)
A048
(1)
A002
(7)
A010
(4)
A019
(2)
A008
(1)
A006
(1)
A037
(2)
A048
(1)
A028
(2)
A001
(44)
A016
(2)
A013
(1)
A051
(1)
A026
(3)
A050
(1)
A009
(3)

f)

AME (n=27)

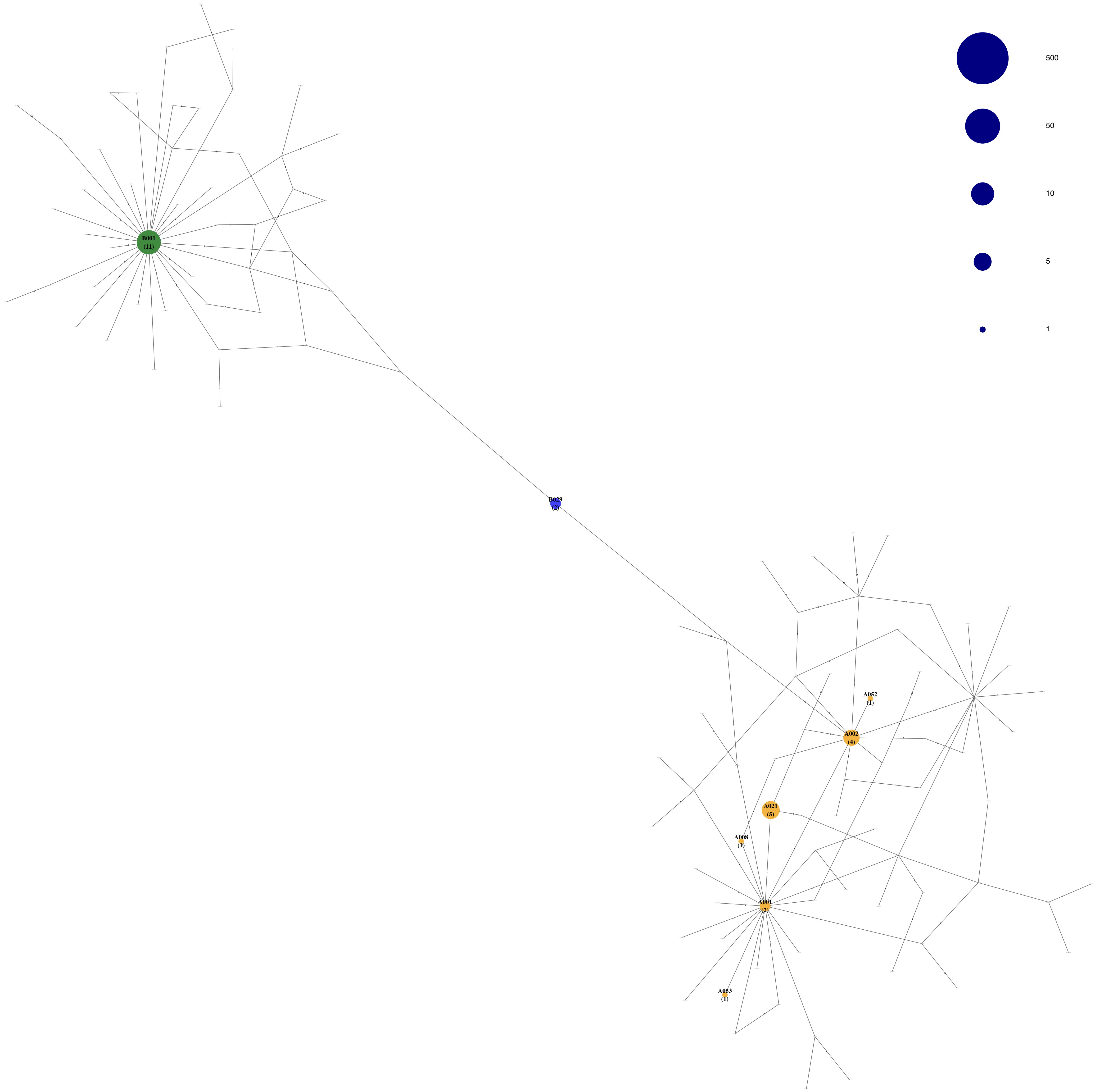
500
50
10
5
1
B001
(11)
B029
(2)
A052
(1)
A002
(4)
A021
(5)
A008
(1)
A001
(2)
A053
(1)

g)

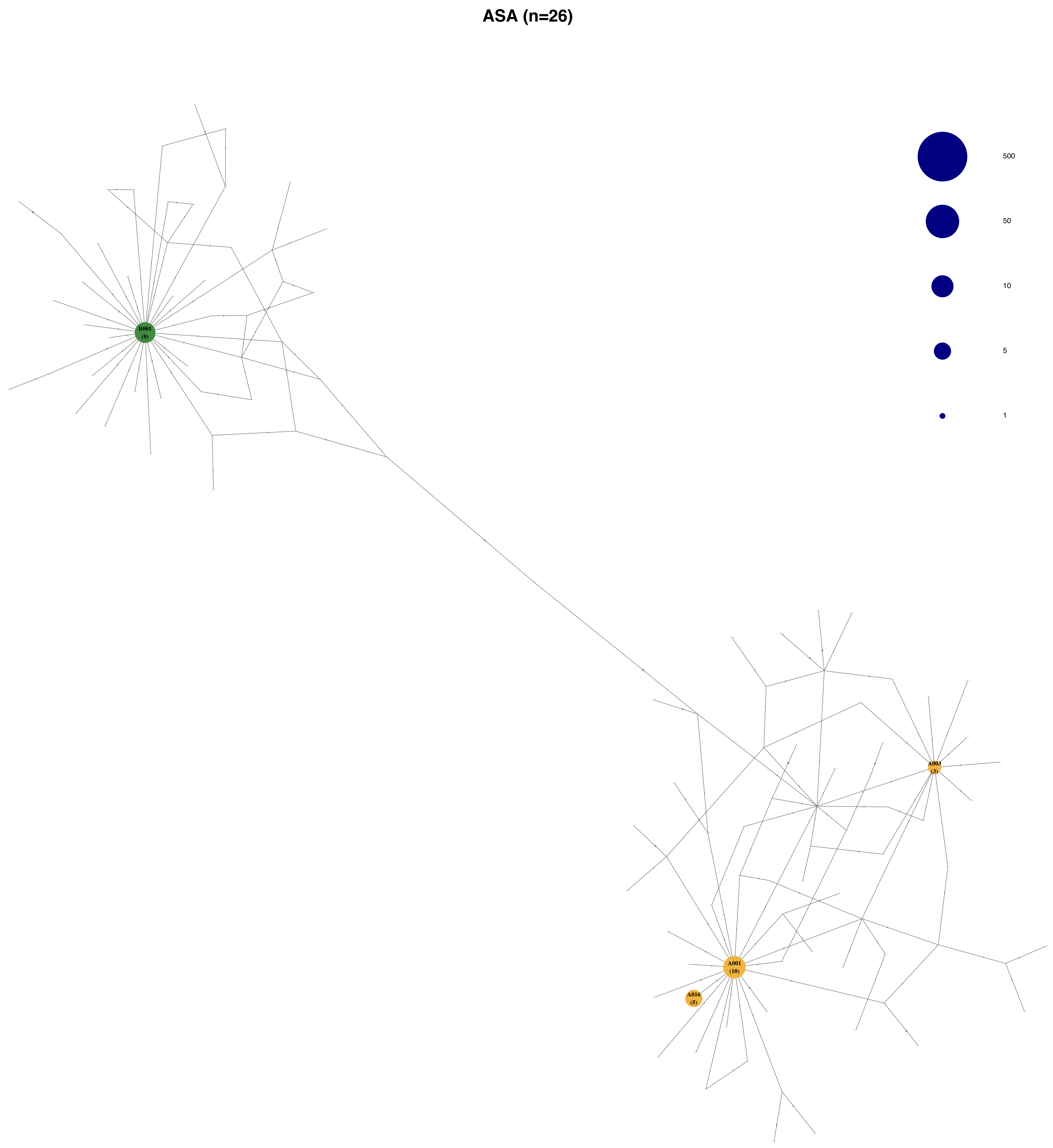

h)

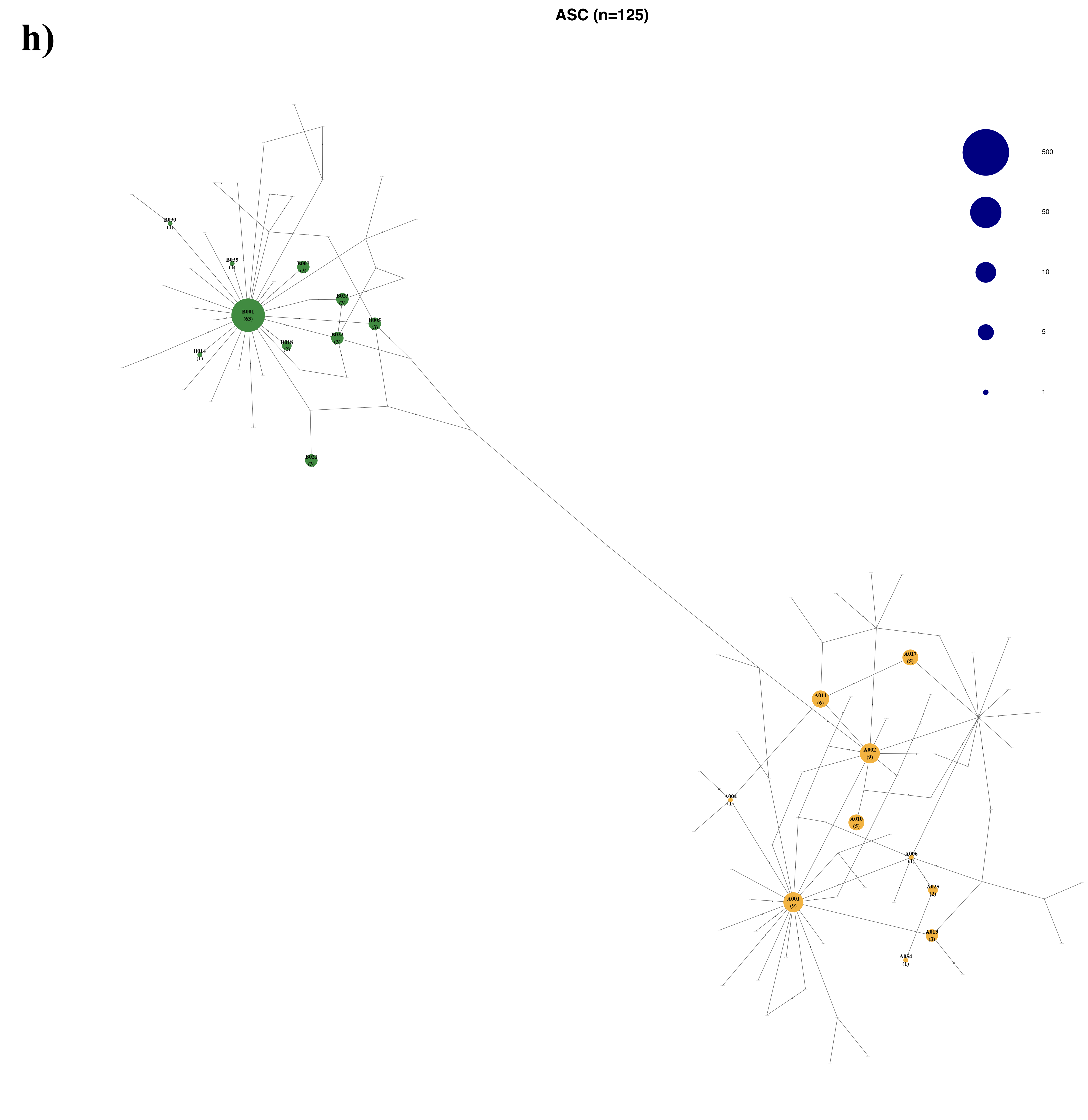
ASC (n=125)
B001
(63)
B030
(1)
B035
(1)
B007
(3)
B023
(3)
B005
(3)
B022
(3)
B018
(2)
B014
(1)
B021
(3)
A017
(5)
A011
(6)
A002
(9)
A004
(1)
A010
(5)
A006
(1)
A025
(2)
A001
(9)
A013
(3)
A054
(1)
500
50
10
5
1

i)

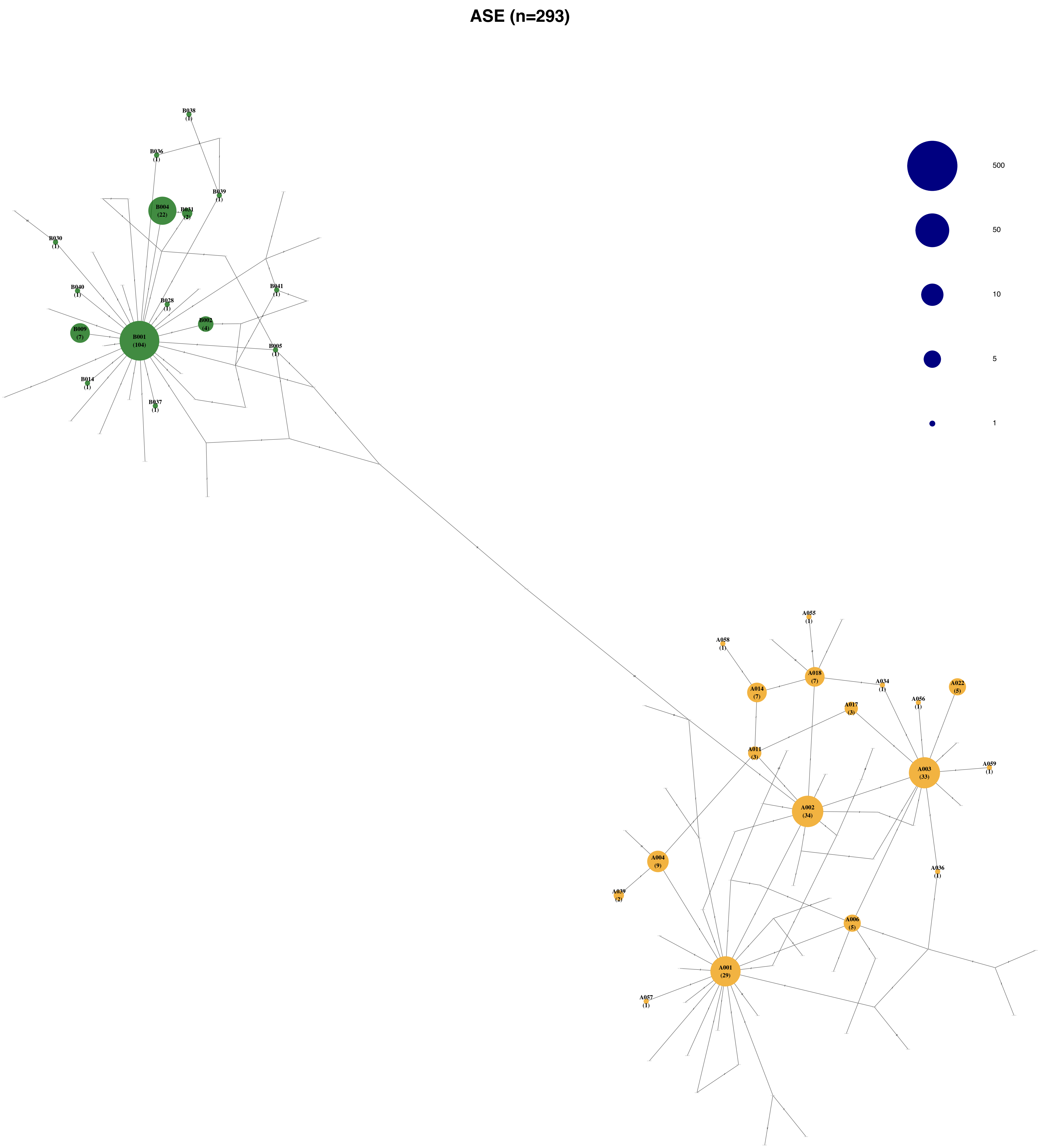
ASE (n=293)
B001
(104)
B004
(22)
B009
(7)
B002
(4)
B031
(3)
B038
(1)
B036
(1)
B039
(1)
B030
(1)
B040
(1)
B028
(1)
B041
(1)
B005
(1)
B014
(1)
B037
(1)
A001
(29)
A002
(34)
A003
(33)
A004
(9)
A006
(5)
A011
(3)
A014
(7)
A017
(3)
A018
(7)
A022
(5)
A034
(1)
A036
(1)
A039
(2)
A055
(1)
A056
(1)
A057
(1)
A058
(1)
A059
(1)
500
50
10
5
1

j)

ASS (n=22)

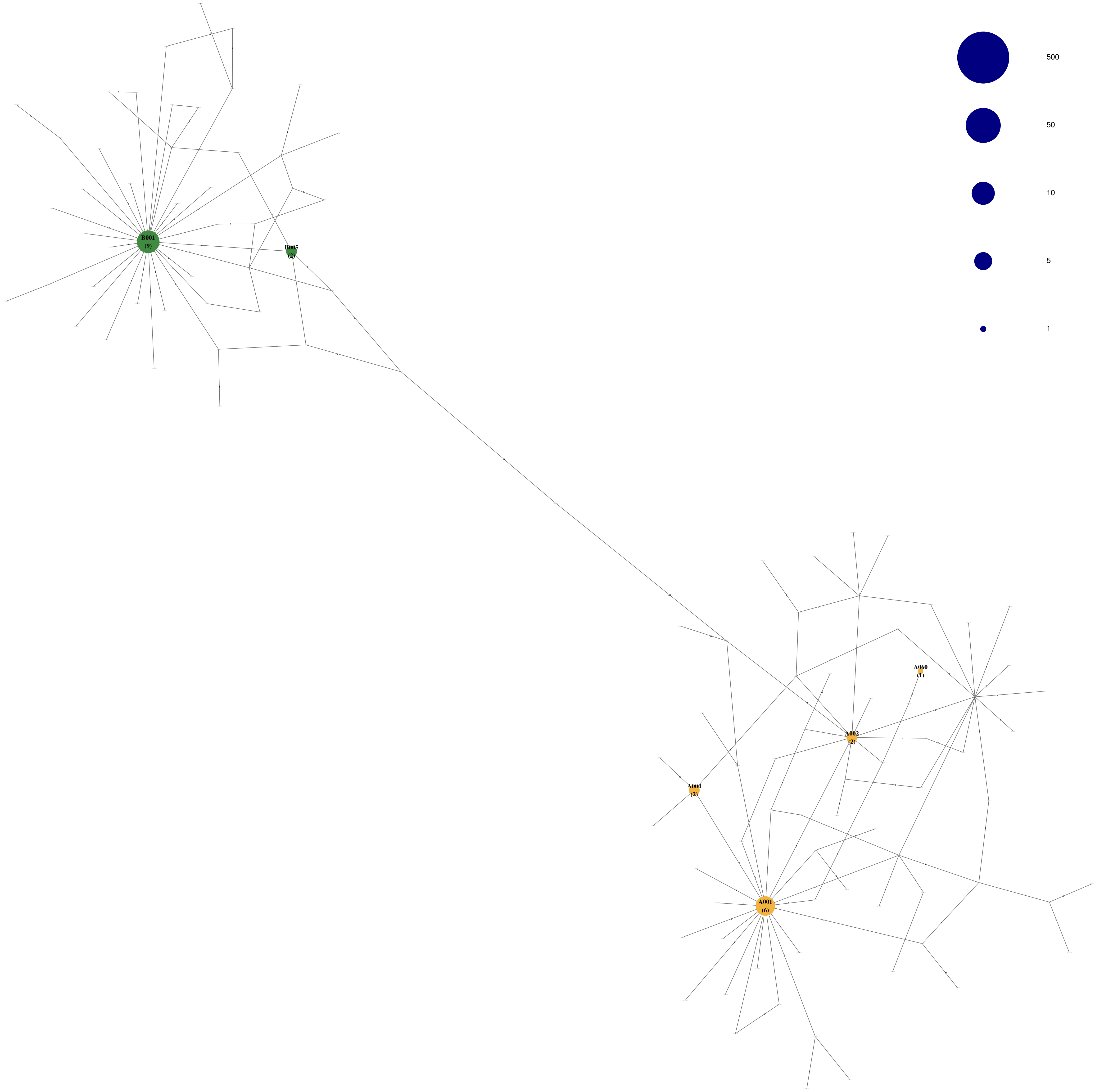

k)

EUB (n=172)

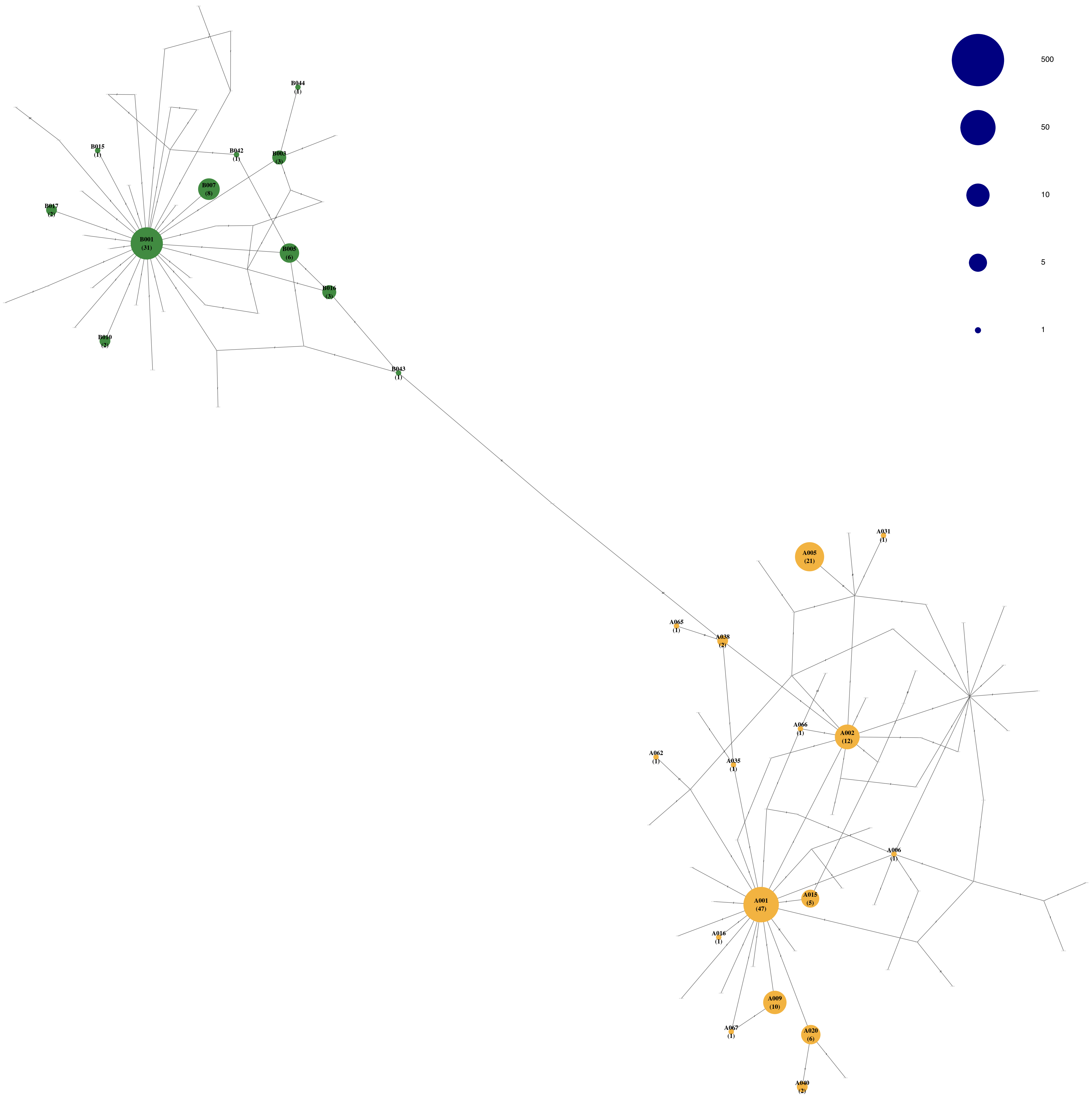
B001
(31)
B007
(8)
B005
(6)
B003
(3)
B016
(3)
B017
(2)
B010
(2)
B015
(1)
B042
(1)
B044
(1)
B043
(1)
A001
(47)
A005
(21)
A002
(12)
A009
(10)
A020
(6)
A015
(5)
A038
(2)
A040
(2)
A031
(1)
A065
(1)
A066
(1)
A062
(1)
A035
(1)
A006
(1)
A016
(1)
A067
(1)
500
50
10
5
1

l)

EUE (n=28)

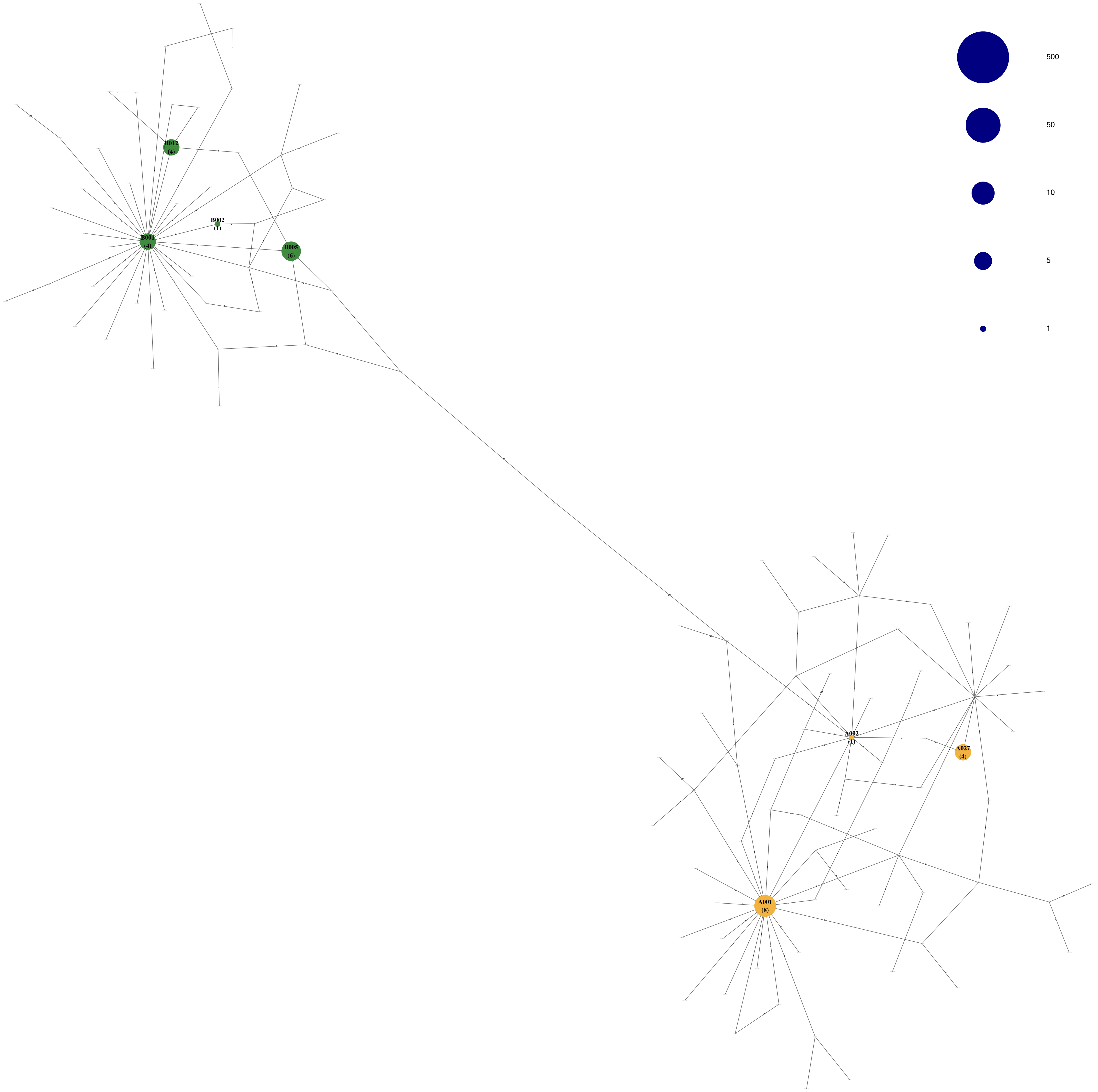

m)

EUI (n=347)

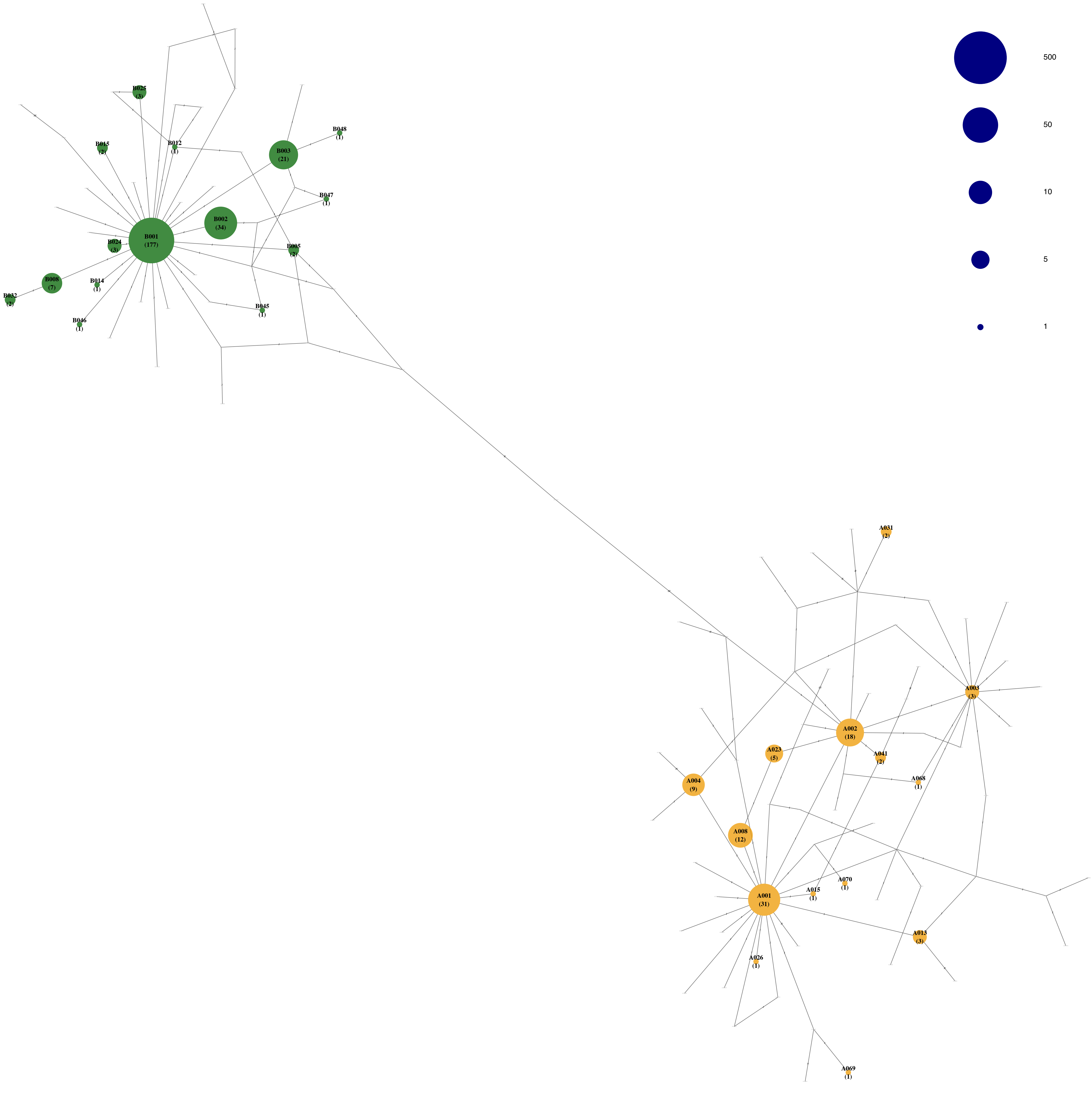
B001 (177)
B002 (34)
B003 (21)
B008 (7)
B024 (3)
B025 (3)
B015 (2)
B005 (2)
B032 (2)
B012 (1)
B048 (1)
B047 (1)
B014 (1)
B045 (1)
B046 (1)
A001 (31)
A002 (18)
A008 (12)
A004 (9)
A023 (5)
A003 (3)
A013 (3)
A031 (2)
A041 (2)
A068 (1)
A070 (1)
A015 (1)
A026 (1)
A069 (1)
500
50
10
5
1

n)

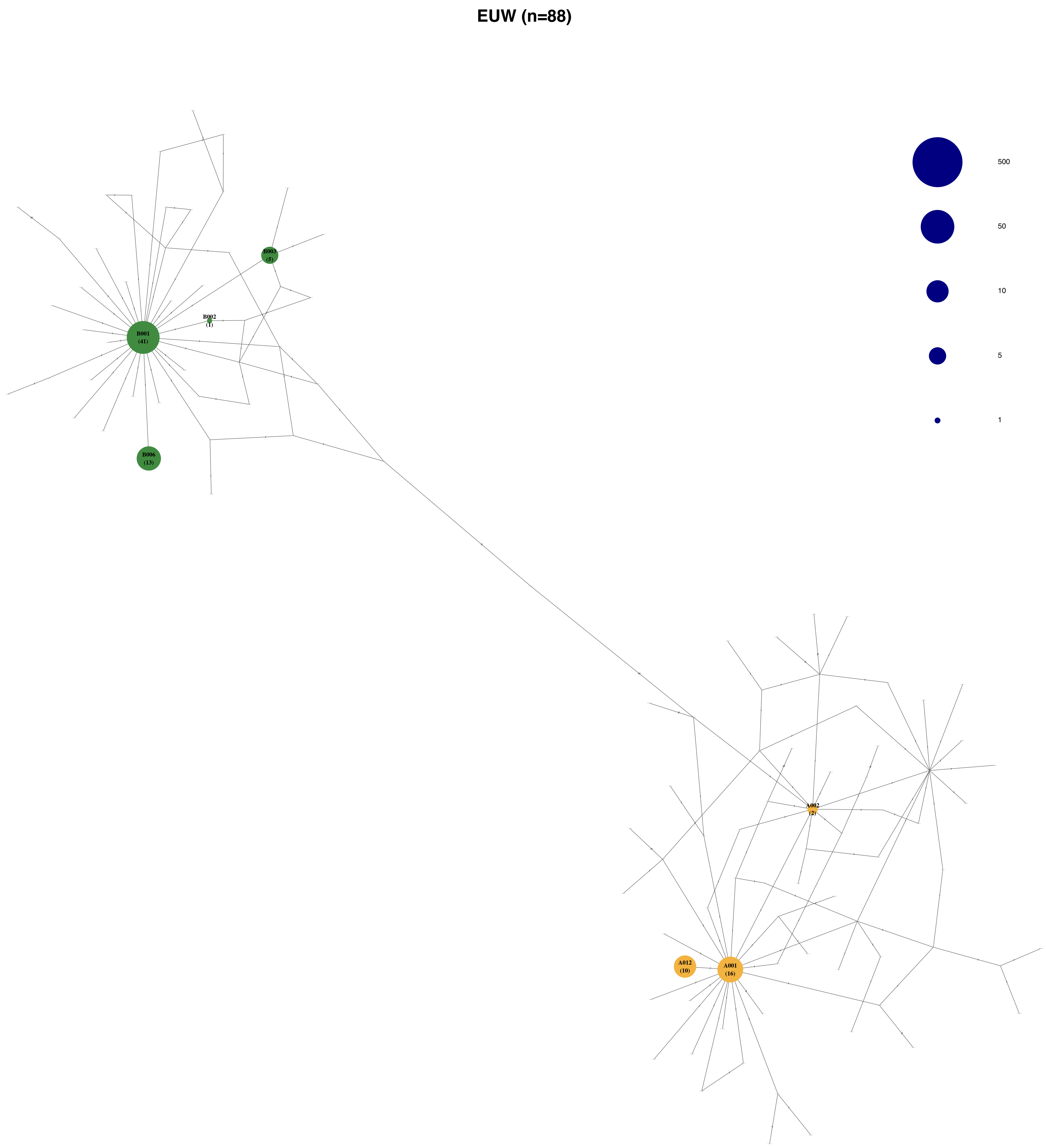
EUW (n=88)
B001
(41)
B002
(1)
B003
(5)
B006
(13)
A001
(16)
A002
(2)
A012
(10)
500
50
10
5
1
