# Supplementary material for: Mitochondrial DNA control-region and coding-region data highlight geographically structured diversity and post-domestication population dynamics in worldwide donkeys
Source: PLoS One. 2024 Aug 28;19(8):e0307511. doi: 10.1371/journal.pone.0307511 (PMC11356394; doi:10.1371/journal.pone.0307511)
Supplement: S1 Text — Custom plot of Network software results in R. (DOCX) [file pone.0307511.s017.docx]

**Additional file 4 Text S1**

**CPNetwork, a custom script to plot Network software results in R**

The Network software (<https://www.fluxus-engineering.com/sharenet.htm>) is a free phylogenetic network software that generates evolutionary trees and networks from genetic, linguistic, and other data.

Sometimes, in particular with complex datasets, the graphical output of the obtained networks is difficult to resolve, with overlapping edges or crowded nodes, and the network customization is tricky.

For these reasons, we are proposing a pipeline to plot Network software results in a highly customizable way called CPNetwork (Custom Plot Network).

**How the pipeline works**

The .fdi file, the final output file from Network software, is firstly parsed with a Python script to extract information on nodes, edges and equivalent taxa files.

Secondly, the files containing the extracted information are used in an R script to plot the custom network.

**Where to find the pipeline**

The pipeline and the technical explanations about CPNetwork are located on GitHub: <https://github.com/marcomilanesi/CPNetwork>

**Parsing**

The CPNetwork_parseFDI.py script is a Python script (version >3) created to parse the .fdi file from Network software.

**Custom plot**

The CPNetwork_pieNetwork.R and CPNetwork_plotNetwork.R scripts are R scripts created to plot a custom network. In the first case, pie charts are used to represent the OUT.

The Igraph R package (version >= 1.1.2) [72] is used.
