# Supplementary material for: Mitochondrial DNA control-region and coding-region data highlight geographically structured diversity and post-domestication population dynamics in worldwide donkeys
Source: PLoS One. 2024 Aug 28;19(8):e0307511. doi: 10.1371/journal.pone.0307511 (PMC11356394; doi:10.1371/journal.pone.0307511)
Supplement: S6 Fig — The picture shows the backbone of the median-joining network depicted in Fig 2 with the alphanumeric codes of control-region haplotypes corresponding to the nodes. (PDF) [file pone.0307511.s014.pdf]

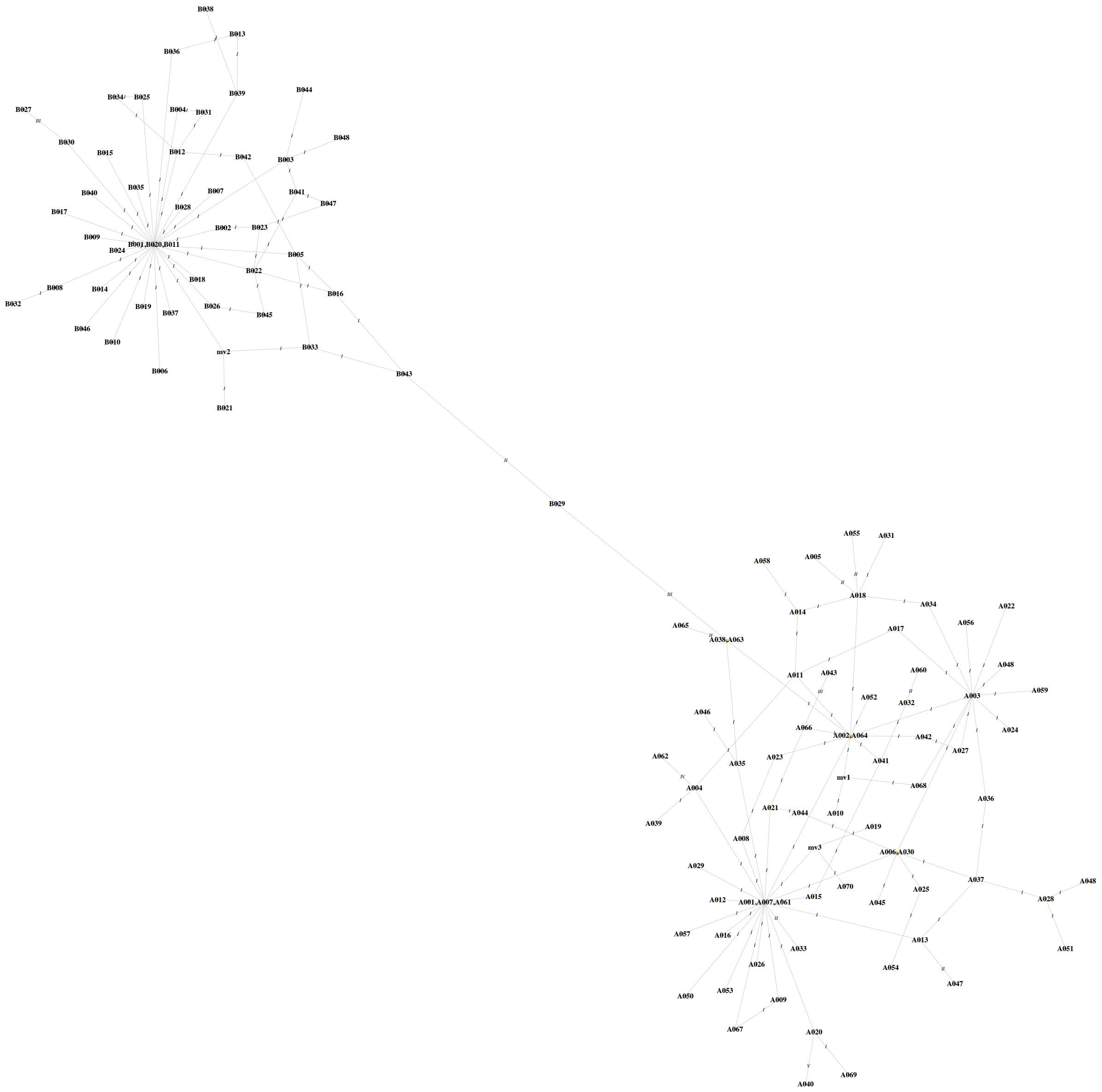

Additional file 12 Figure S6. Median-joining network backbone with control-region haplotype labelling.
